# Fruit-localized phytochromes regulate plastid biogenesis, starch synthesis, and carotenoid metabolism in tomato

**DOI:** 10.1093/jxb/ery145

**Published:** 2018-04-18

**Authors:** Ricardo Ernesto Bianchetti, Bruno Silvestre Lira, Scarlet Santos Monteiro, Diego Demarco, Eduardo Purgatto, Christophe Rothan, Magdalena Rossi, Luciano Freschi

**Affiliations:** 1 Departamento de Botânica, Instituto de Biociências, Universidade de São Paulo, Rua do Matão, São Paulo, Brazil; 2 Departamento de Alimentos e Nutrição Experimental, Faculdade de Ciências Farmacêuticas, Universidade de São Paulo, Av. Professor Lineu Prestes, São Paulo, Brazil; 3 INRA, Université de Bordeaux, UMR 1332 Biologie du Fruit et Pathologie, Villenave d’Ornon, France

**Keywords:** Auxin, carotenoid, cytokinin, fleshy fruit, phytochrome, plastid division, tomato, *Solanum lycopersicum*, starch

## Abstract

Light signaling has long been reported to influence fruit biology, although the regulatory impact of fruit-localized photoreceptors on fruit development and metabolism remains unclear. Studies performed in phytochrome (PHY)-deficient tomato (*Solanum lycopersicum*) mutants suggest that SlPHYA, SlPHYB2, and to a lesser extent SlPHYB1 influence fruit development and ripening. By employing fruit-specific RNAi-mediated silencing of *SlPHY* genes, we demonstrated that fruit-localized SlPHYA and SlPHYB2 play contrasting roles in regulating plastid biogenesis and maturation in tomato. Our data revealed that fruit-localized SlPHYA, rather than SlPHYB1 or SlPHYB2, positively influences tomato plastid differentiation and division machinery via changes in both light and cytokinin signaling-related gene expression. Fruit-localized SlPHYA and SlPHYB2 were also shown to modulate sugar metabolism in early developing fruits via overlapping, yet distinct, mechanisms involving the co-ordinated transcriptional regulation of genes related to sink strength and starch biosynthesis. Fruit-specific *SlPHY* silencing also drastically altered the transcriptional profile of genes encoding light-repressor proteins and carotenoid-biosynthesis regulators, leading to reduced carotenoid biosynthesis during fruit ripening. Together, our data reveal the existence of an intricate PHY–hormonal interplay during fruit development and ripening, and provide conclusive evidence on the regulation of tomato quality by fruit-localized phytochromes.

## Teaser

Fruit-localized phytochromes and their downstream signaling cascades not only modulate chloroplast biogenesis in immature tomato fruits but also regulate sugar and carotenoid accumulation, two essential features of tomato fruit quality.

## Introduction

Fleshy fruit growth, maturation, and ripening are under strict developmental, hormonal, and epigenetic regulation, which in turn are fine-tuned by a plethora of environmental stimuli (Kumar *et al.*, 2014; Giovannoni *et al.*, 2017). Among environmental cues, light plays a significant role in determining fruit growth, pigmentation, and timing of ripening (Carvalho *et al.*, 2011; Gupta *et al.*, 2014; Llorente *et al.*, 2016*a*). In tomato (*Solanum lycopersicum*), a major crop and important model species for fleshy fruits, several lines of evidence indicate that changes in light perception and signaling can lead to significant alterations in fruit development and quality traits (Giliberto *et al.*, 2005; Schofield and Paliyath, 2005; Azari *et al.*, 2010*b*; Bianchetti *et al.*, 2017).

One of the earliest pieces of evidence of the influence of light on tomato fruit biology dates back to 1954, when fruit pigmentation was shown to be regulated by red/far red (R/FR) light in a reversible manner (Piringer and Heinze, 1954). First isolated only a few years later, phytochromes (PHYs) act as molecular switches in response to R and FR light, existing as homodimers of two independently reversible subunits. Once activated by R light, PHYs are transported from the cytosol to the nucleus, where they counteract light-signaling repressor proteins, such as CONSTITUTIVE PHOTOMORPHOGENESIS1 (COP1), CULLIN4 (CUL4), DNA DAMAGE-BINDING PROTEIN 1 (DDB1), DETIOLATED1 (DET1), and PHYTOCHROME INTERACTION FACTOR (PIF) (Deng and Quail, 1992; Pepper *et al.*, 1994; Schroeder *et al.*, 2002; Duek and Fankhauser, 2005; Thomann *et al.*, 2005). In line with their role as repressors of photomorphogenic responses, either the down-regulation or loss-of-function of tomato genes encoding COP1, CUL4, DDB1, DET1, and PIF1a profoundly alter tomato fruit physiology and nutritional composition (Cookson *et al.*, 2003; Liu *et al.*, 2004; Davuluri *et al.*, 2005; Kolotilin *et al.*, 2007; Wang *et al.*, 2008; Azari *et al.*, 2010*b*; Enfissi *et al.*, 2010; Llorente *et al.*, 2016*b*).

In tomato, five PHY-encoding genes have been identified, namely *SlPHYA*, *SlPHYB1*, *SlPHYB2*, *SlPHYE*, and *SlPHYF* (Alba *et al.*, 2000*b*). The paralogous *SlPHYB1* and *SlPHYB2*, which originated during the *Solanum* whole-genome triplication event (Tomato Genome Consortium, 2012), display distinct expression profiles within tomato organs, pointing to functional diversification (Hauser *et al.*, 1997; Weller *et al.*, 2000). *SlPHYB1* is more prominently expressed in vegetative tissues, whereas the highest *SlPHYB2* expression levels are detected in fruits (Hauser *et al.*, 1997; Bianchetti *et al.*, 2017). Moreover, evidence also suggests a more direct involvement of *SlPHYB1*, rather than *SlPHYB2*, during early seedling photomorphogenic responses (van Tuinen *et al.*, 1995*a*, 1995*b*; Weller *et al.*, 2000). Very little is known about the influence of *SlPHYE* and *SlPHYF* on tomato vegetative and reproductive development (Schrager-Lavelle *et al.*, 2016).

Attempts to define the influence of fruit-localized PHYs on fruit development and ripening have been relatively limited. Brief R-light treatments of detached mature-green tomato fruits promote lycopene accumulation, a response reversed by subsequent treatment with FR light (Alba *et al.*, 2000*a*), which is consistent with the hypothesis that fruit-localized PHYs play a regulatory role in controlling tomato fruit carotenogenesis. The marked accumulation of *SlPHYA* transcripts during fruit ripening (Alba *et al.*, 2000*a*) associated with the reduced fruit lycopene levels observed in *phyA* tomato mutants (Gupta *et al.*, 2014) raise the possibility that this PHY may be an important regulator of tomato fruit carotenoid biosynthesis. However, regardless of the development stage or tissue considered, *SlPHYB2* is the most highly expressed *PHY* in tomato fruits (Bianchetti *et al.*, 2017). Moreover, the *phyB2* mutant also displays considerable changes in the fruit carotenoid profile (Gupta *et al.*, 2014), suggesting that multiple PHYs are involved in regulating this metabolic process.

Besides carotenogenesis, PHYs have also been found to control other aspects of tomato fruit development and metabolism, including chloroplast biogenesis, chlorophyll accumulation, sugar metabolism, sink activity, and hormonal signaling (Gupta *et al.*, 2014; Bianchetti *et al.*, 2017). However, as the existing evidence supporting these findings is exclusively based on studies performed in *phy* mutants, whether these responses are dependent on fruit-localized PHYs or are merely consequences of the collateral negative effects of PHY deficiency on vegetative plant growth remains to be elucidated.

By employing fruit-specific RNAi-mediated silencing of *SlPHY* genes, we shed light on the functional specificity of fruit-localized SlPHYs in controlling developmental and metabolic processes associated with sugar and carotenoid accumulation, two essential nutritional quality traits of this edible fruit. Our data also reveal that an intricate light–hormonal signaling network involving key components of both auxin and cytokinin signal transduction pathways is implicated in the PHY-dependent regulation of fruit plastid biogenesis, sugar metabolism, and carotenoid accumulation.

## Materials and methods

### Plant material and growth conditions

Tomato (*Solanum lycopersicum* L.) plants cv. Micro-Tom, which harbors the wild-type *SlGLK2* allele (Carvalho *et al.*, 2011), were grown under controlled conditions of 250 µmol m^−2^ s^−1^, a 12-h photoperiod, and air temperature of 27/22 °C day/ night. The fruit stages examined were immature green, mature green, breaker, and red ripe, which were harvested on average at 8, 25, 32, and 44 d post-anthesis. All fruits were harvested at the same time of the day with four biological replicates (each replicate was composed of a pool of at least five fruits from different plants). Columella, placenta, and seeds were immediately removed, and the remaining tissues were frozen in liquid nitrogen and stored at –80 °C until use.

### Generation of transgenic tomato plants

Three fragments specific to the coding sequences of *SlPHYA*, *SlPHYB2*, and both *SlPHYB1* and *SlPHYB2* were selected using BLAST queries against the Sol Genomics Network database (https://solgenomics.net/, ITAG release 2.40) and the web-based computational tool pssRNAit (Dai and Zhao, 2011) was employed to avoid off-target silencing. Each fragment was independently cloned into pENTR D-TOPO plasmids (Invitrogen) using the primers listed in Supplementary Table S1 at *JXB* online. Subsequently, each fragment was recombined into the plant transformation vector pK8GWIWG (Fernandez *et al.*, 2009). Transgenic Micro-Tom plants were generated by *Agrobacterium*-mediated transformation according to Pino *et al.* (2010), with minor changes: cotyledons from 5-d-old seedlings were used for the transformation, and the zeatin and kanamycin concentration were 5 µM and 70 mg l^−1^, respectively. All plants used in the study were from the T_2_ generation.

### Fruit color and pigment quantification

Changes in fruit color (Hue angle) were determined using a Konica Minolta CR-400 colorimeter as described in Su *et al.* (2015). Chlorophyll extraction and quantification were carried out as described in Lira *et al.* (2016) with some modifications. Pericarp samples were weighed (typically 100 mg fresh weight, FW), ground in liquid nitrogen, immersed in a 10× excess volume of *N*, *N*-dimethylformamide, and incubated at room temperature for 24 h in absolute darkness and constant agitation (200 rpm). After centrifugation (9000 *g*, 5 min, 4 °C), the supernatant absorbance was recorded at 647 and 664 nm, and the total chlorophyll content was estimated using the equations given by Porra *et al.* (1989).

For carotenoid extraction, approximately 200 mg FW of pericarp samples were ground in liquid nitrogen and sequentially homogenized with a solution of 100 µl of saturated NaCl, then 200 µl of dichloromethane, and finally 1 ml of hexane:diethyl ether (1:1, v/v). The supernatant was collected after centrifugation (5000 *g*, 10 min, 4 °C). The remaining carotenoids in the pellet were extracted three more times with 500 µl of hexane:diethyl ether (1:1, v/v). All supernatant fractions were combined, completely vacuum-dried, and suspended with 200 µl of acetonitrile. Lycopene, β-carotene, lutein, and neurosporene levels were determined by high-performance liquid chromatography (HPLC) with a photodiode array detector (PDA) as described by Lira *et al.* (2017).

### Starch and soluble sugar quantification

Starch and soluble sugar extractions were performed as described in Bianchetti *et al.* (2017). Briefly, approximately 200 mg FW of pericarp samples was extracted with 1 ml of 80% (v/v) methanol for 10 min at 80 °C followed by the collection of the supernatants by centrifugation (13000 *g*, 10 min, 4 °C). The remaining pellets were re-extracted five times, and all supernatants were combined, completely vacuum-dried, and suspended in 200 µl distilled water. Soluble sugars (i.e. sucrose, fructose, and glucose) were measured using a HPLC system equipped with an amperometric detector (Dionex, Sunnyvale, USA) and a CarboPac PA1 (4 × 250 mm) column (Purgatto *et al.*, 2002). Starch levels were determined from dried pellet as described in Suguiyama *et al.* (2014).

### Antioxidant capacity and total phenolics

Hydrophilic and lipophilic Trolox equivalent antioxidant capacities (TEACs) were spectrophotometrically determined as described in Lira *et al.* (2016). Total phenolic content was determined in hydrophilic extracts by using the Folin–Ciocalteu method (Singleton and Rossi, 1965).

### Plastid ultrastructure and abundance

Pericarp fragments taken from the pedicel region (green shoulder) of immature fruits were fixed at 4 °C in 2.5% (v/v) glutaraldehyde and 2% (v/v) paraformaldehyde in 0.1 M sodium phosphate buffer (pH 7.2). Subsequently, the samples were post-fixed in 1% osmium tetroxide in 0.1 M sodium phosphate buffer (pH 7.2), dehydrated in a graded acetone series, and embedded in Spurr’s resin. Ultrathin sections were stained with saturated uranyl acetate and lead citrate (Melo *et al.*, 2016) and observed using a JEOL JEM1011 transmission electron microscope. Sections from three immature fruits picked from different plants were analysed per genotype.

Plastid abundance was determined as described in Bianchetti *et al.* (2017). Briefly, small pieces (1 × 1 mm) of pericarp were fixed in 3.5% (v/v) glutaraldehyde for 1 h. Samples were washed twice and transferred to 0.1 M NaEDTA pH 9.5 solution for 4 h at 60 °C in complete darkness. Pieces were softly disrupted and transferred to microscope slides. Isolated cells were visualized using a Leica microscope. Plastid densities in individual cells were estimated using the ImageJ program (https://imagej.nih.gov/ij/). At least 40 individual cells were analysed per sample.

### Transcriptional profile

Total RNA extraction, cDNA synthesis, primer design, and qPCR assays were performed as described by Quadrana *et al.* (2013). Primer sequences used are detailed in Supplementary Table S1. Quantitative real-time (qRT-)PCR reactions were performed in a StepOnePlus PCR Real-Time thermocycler (Applied Biosystems) in a final volume of 10 µl using 2× SYBR Green Master Mix reagent (Thermo Fisher Scientific). Melting curves were checked for unspecific amplifications and primer dimerization. Absolute fluorescence data were analysed using the LinRegPCR software package (Ruijter *et al.*, 2009) to obtain quantitation cycle (*C*_q_) values and to calculate primer efficiency. Transcript abundances were normalized against the geometric mean of two reference genes, *CAC* and *EXPRESSED* (Expósito-Rodriguez *et al*., 2008).

### Gene promoter analysis

Gene promoter analysis was performed using the promotor sequences available at the Sol Genomics Network. Typically, 3 kb upstream of the initial ATG codon of each sequence was analysed using the PlantPAN 2.0 platform (http://plantpan2.itps.ncku.edu.tw/) (Chow *et al.*, 2016) for the presence of PBE-box (CACATG), G-box (CACGTG), CA-hybrid (GACGTA), CG-hybrid (GACGTG), canonical AuxRE (TGTGTC), and degenerate AuxRE (TGTGNC) motifs (Martínez-García *et al.*, 2000; Song *et al.*, 2008; Chaabouni *et al.*, 2009).

### Statistical analysis

ANOVA and Student’s *t*-test were performed using the JMP statistical software package (14th edition; http://jmp.com). Comparisons with *P*<0.05 were considered statistically significant. Data from wild-type and all independent transgenic lines were also compared with principal component analysis (PCA) using the InfoStat software (http://infostat.com.ar).

## Results

### Fruit-specific *PHY* knockdown in transgenic tomato plants

**Fig. 1.**
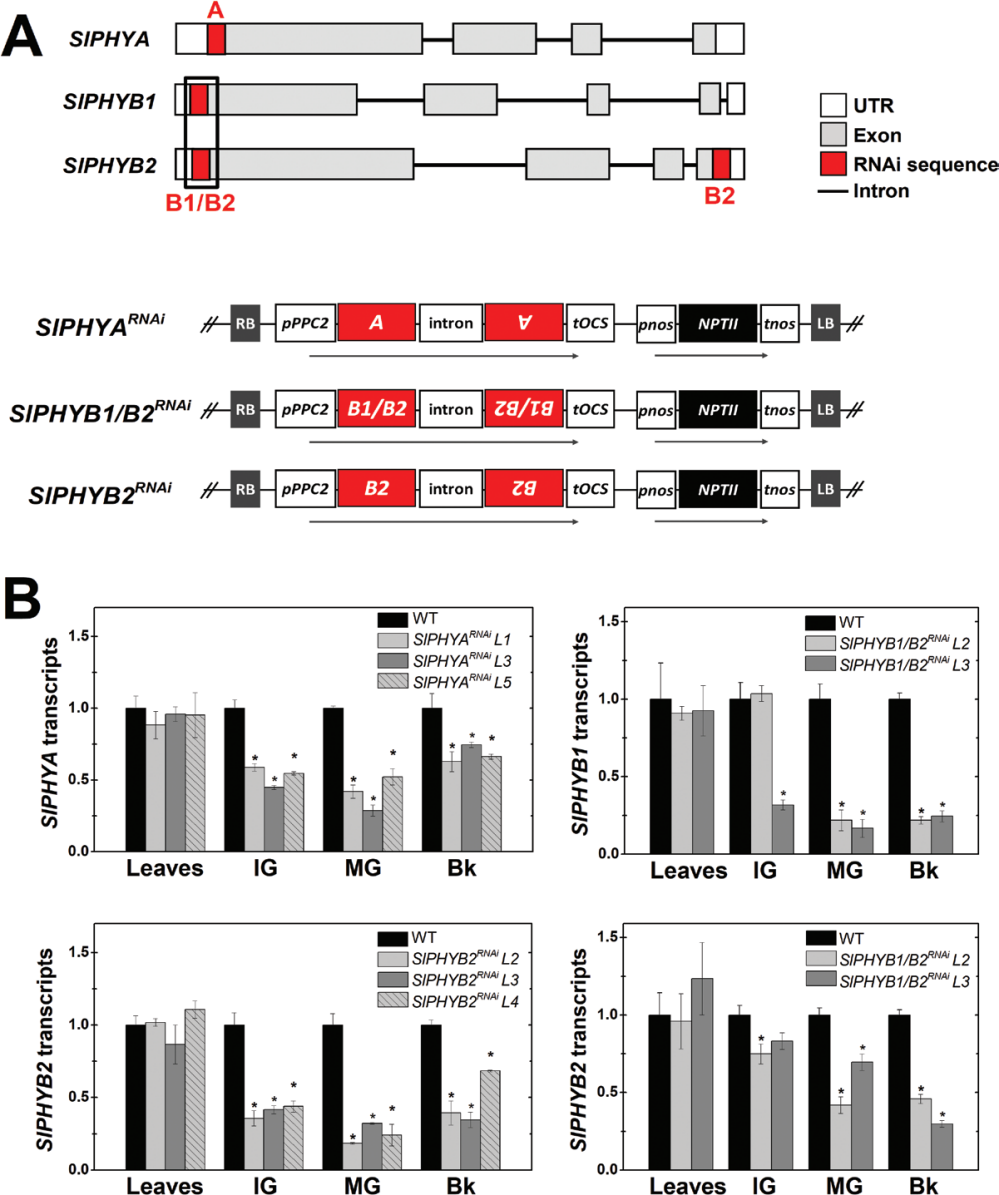
Fruit-specific *PHY* knockdown in transgenic tomato plants. (A) Constructs designed for generation of the *SlPHYA*^*RNAi*^, *SlPHYB1/B2*^*RNAi*^, and *SlPHYB2*^*RNAi*^ transgenic lines. ‘A’ indicates the *SlPHYA*-specific fragment of the mRNA 5′ untranslated region (UTR). ‘B1/B2’ indicates the *SlPHYB1/B2*-specific fragment of the mRNA 5′ UTR. ‘B2’ indicates the *SlPHYB2*-specific fragment of the mRNA 5′ UTR. (B) Relative *SlPHY* mRNA levels in leaves, and immature green (IG), mature green (MG), and breaker (Bk) stages of fruits of the *SlPHYA*^*RNAi*^, *SlPHYB2*^*RNAi*^, and *SlPHYB1/B2*^*RNAi*^ lines. The first and second fully expanded leaves from the top of 2-month-old plants were harvested. Transcript abundance was normalized against the wild-type (WT) sample. Statistically significant differences compared with the WT genotype were determined using Student’s *t*-test: *P<0.05. Data are means (±SE) of at least three biological replicates. (This figure is available in color at *JXB* online.)

To investigate the role played by distinct PHYs in tomato fruit development and ripening, we generated fruit-specific silenced tomato plants with reduced mRNA levels of *SlPHYA*, *SlPHYB2*, or both *SlPHYB1* and *SlPHYB2*. This was achieved using a hairpin-mediated RNAi approach based on the expression of specific fragment sequences of these genes under the control of the fruit-specific *PPC2* promoter (Fernandez *et al.*, 2009). The transgenic plants obtained, hereafter designated as *SlPHYA*^*RNAi*^, *SlPHYB2*^*RNAi*^, and *SlPHYB1/B2*^*RNAi*^ (Fig. 1A), were generated in a Micro-Tom background homozygous for the wild-type *GOLDEN2-LIKE-2* (*SlGLK2*) allele (Carvalho *et al.*, 2011), which encodes a transcription factor critically important for chloroplast development in tomato fruits (Powell *et al.*, 2012).

Transcript abundance analysis revealed that *SlPHYA*, *SlPHYB2*, and both *SlPHYB1* and *SlPHYB2* were down-regulated in the *SlPHYA*^*RNAi*^, *SlPHYB2*^*RNAi*^, and *SlPHYB1/B2*^*RNAi*^ lines, respectively (Fig. 1B). A search for potential tomato off-targets via BLAST queries against the Sol Genomics Network database or via the public web-based computational tool pssRNAit (Dai and Zhao, 2011) failed to identify regions in the tomato coding that exhibited the 21-nucleotide perfect identity threshold reported to cause off-target silencing (Xu *et al.*, 2006). The percentage of identity of the silencing fragments was below 60% with non-target tomato *PHY* genes (Supplementary Table S2). Moreover, the length of stretches with perfect identity between the RNAi fragments and non-target tomato *PHY* genes was ≤15 nucleotides (Supplementary Table S2). In line with this, no off-target *SlPHY* silencing was detected in the transgenic lines generated (Supplementary Fig. S1).

In all the transgenic lines, *PHY* knockdown was restricted to the fruit tissues as no significant *PHY* silencing was observed in leaf samples (Fig. 1B). Transgenic lines exhibited normal plant growth and visual phenotypic features similar to those found in wild-type (WT) plants (Supplementary Fig. S2). Overall, fruit-specific *PHY* knockdown caused no marked changes in fruit size and ripening progression (Supplementary Fig. S3).

### Fruit-localized SlPHYA and SlPHYB2 differentially impact chloroplast biogenesis and differentiation during early fruit development

**Fig. 2.**
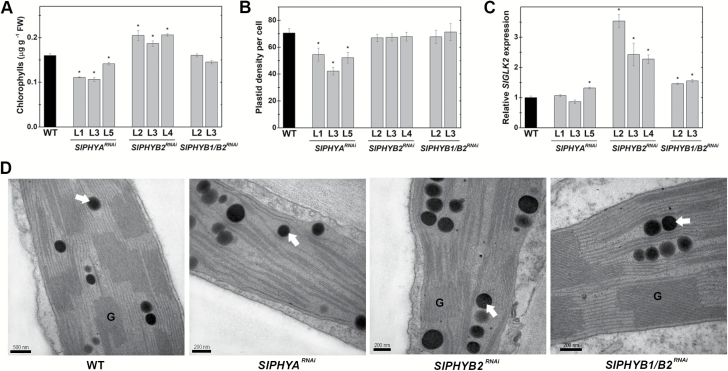
Fruit-localized SlPHYA and SlPHYB2 differentially impact on chloroplast biogenesis and differentiation during early fruit development. (A) Total chlorophyll content in immature fruits. (B) Plastid abundance per pericarp cell of immature fruits. (C) Relative mRNA levels of *GOLDEN2-LIKE-2* (*SlGLK2*) normalized against the wild-type (WT) sample. Statistically significant differences compared with the WT sample were determined using Student’s *t*-test: *P<0.05. Chlorophyll content and transcript abundance data are means (±SE) of at least three biological replicates. For plastid density, three fruits of each genotype were randomly picked, and two technical replicates were taken at the pedicel region of each fruit. Plastid density was determined in at least 40 individual cells per sample. (D) Representative TEM images of plastids in the pedicel region of immature fruits. Arrows indicate plastoglobuli. G, granal thylakoid.

The PHY-dependent regulation of chloroplast development has been extensively reported in leaf tissues of several species (Stephenson *et al.*, 2009; Inagaki *et al.*, 2015). Moreover, some recent reports have also indicated altered chlorophyll accumulation and chloroplast biogenesis in immature fruits of PHY-deficient tomato mutants (Gupta *et al.*, 2014; Bianchetti *et al.*, 2017). Compared to the WT, fruit-specific *SlPHYA* and *SlPHYB2* knockdown reduced and increased the chlorophyll content in immature fruits, respectively (Fig. 2A). However, chlorophyll levels in immature fruits from *SlPHYB1/B2*^*RNAi*^ plants were similar to WT counterparts.

Microscopy analysis of pericarp cells revealed that the reduced chlorophyll content detected in *SlPHYA*^*RNAi*^ immature fruits was associated with a reduction of up to 40% in the number of chloroplasts per pericarp cell compared to WT fruits (Fig. 2B). However, the higher chlorophyll content observed in *SlPHYB2*^*RNAi*^ immature fruits was not accompanied by changes in plastid abundance but instead was linked to the up-regulation of the master regulator of chloroplast development and maintenance, *SlGLK2* (Fig. 2C). *SlPHYB1/B2* knockdown lines showed an intermediate impact on fruit chlorophyll content, plastid density, and *SlGLK2* mRNA levels, exhibiting unaltered chlorophyll levels and chloroplast abundance in pericarp cells and slightly higher expression of *SlGLK2* compared to the WT (Fig. 2).

Plastids of WT, *SlPHYB2*^*RNAi*^, and *SlPHYB1/B2*^*RNAi*^ immature fruits exhibited remarkably similar internal membranous structures, displaying well-developed grana and stroma thylakoids as well as numerous plastoglobuli (Fig. 2D, Supplementary Fig. S4). In contrast, fruit-specific *SlPHYA* knockdown resulted in the formation of chloroplasts with highly reduced grana, suggesting a promotive role of PHYA-mediated light perception on fruit plastid granal development. Plastoglobuli and starch grains were observed equally in fruit chloroplasts of the WT and all transgenic lines.

**Fig. 3.**
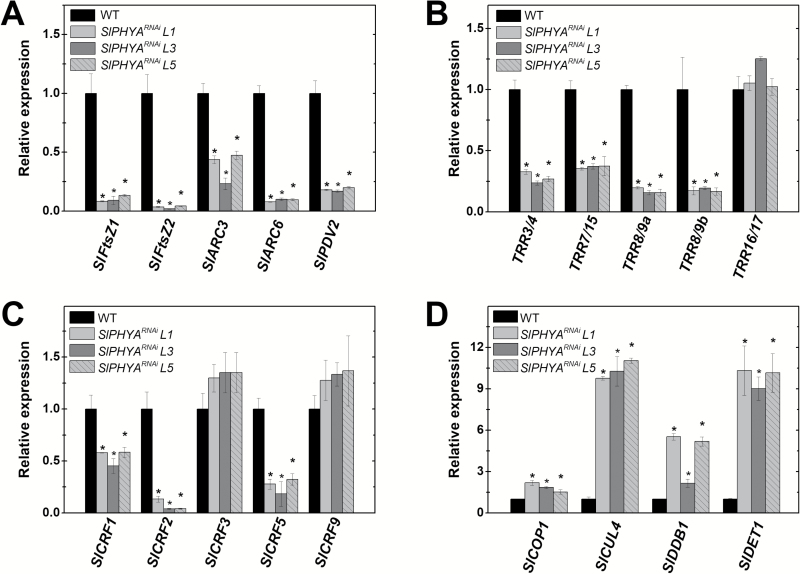
SlPHYA-mediated regulation of chloroplast division machinery is associated with changes in the transcript abundance of light- and cytokinin-signaling genes. (A) Relative mRNA levels of genes encoding components of the plastid division machinery in immature fruits. (B) Relative mRNA levels of type-A *TOMATO RESPONSE REGULATOR* (*TRR*) genes in immature fruits. (C) Relative mRNA levels of *CYTOKININ RESPONSE FACTOR* (*SlCRF*) genes in immature fruits. (D) Relative mRNA levels of genes encoding light-signaling repressor proteins. Data are means (±SE) of at least three biological replicates. Transcript abundance was normalized against the wild-type (WT) sample. Statistically significant differences compared with the WT were determined using Student’s *t*-test: *P<0.05). FtsZ, filamentous temperature sensitive-Z; ARC, accumulation and replication of chloroplasts; PDV2, plastid division 2; COP1, constitutive photomorphogenic 1; CUL4, cullin 4; DDB1, UV-damaged DNA binding protein 1; DET1, de-etiolated1.

As neither *SlPHYB2* nor the *SlPHYB1/B2* knockdown altered chloroplast density per cell or plastid ultrastructure (Fig. 2), fruit-localized SlPHYA seems to play a preponderant role in controlling chloroplast biogenesis and differentiation in early developing fruits. Transcript abundance analysis revealed that the reduced plastid abundance observed in *SlPHYA*-silenced fruits was most probably explained by a drastic reduction in mRNA levels of genes encoding key components of the plastid division machinery, such as FILAMENTOUS TEMPERATURE SENSITIVE-Z (FtsZs), ACCUMULATION AND REPLICATION OF CHLOROPLASTS (ARCs), and PLASTID DIVISION 2 (PDV2), compared to the WT genotype (Fig. 3A).

Given the key role played by cytokinins in regulating plastid division and maturation in plants and the widely reported crosstalk between this hormonal class and PHY signaling (Okazaki *et al.*, 2009; Cortleven and Schmülling, 2015), a transcriptional profiling of type-A *TOMATO RESPONSE REGULATOR* (*TRR*) was performed. Four out of the five type-A *TRR*s analysed were significantly down-regulated in immature fruits of *SlPHYA*^*RNAi*^ compared to the WT genotype (Fig. 3B). Moreover, among the five *CYTOKININ RESPONSE FACTOR* genes most highly expressed in tomato fruit tissues (Shi *et al.*, 2012), *SlCRF1*, *SlCRF2*, and *SlCRF5* were markedly down-regulated in *SlPHYA*^*RNAi*^ lines, whereas *SlCRF3* and *SlCRF9* mRNA levels remained unchanged (Fig. 3C). As *AtCRF2* is responsible for inducing *AtPDV2*, subsequently increasing plastid division rates in Arabidopsis (Okazaki *et al.*, 2009), the drastic down-regulation of both *SlCRF2* and *SlPDV2* in *SlPHYA*-silenced fruits suggests that a similar regulatory mechanism also takes place early in the development of tomato fruits.

Alongside the down-regulation of cytokinin signaling genes, fruit-specific *SlPHYA*-silencing resulted in the up-regulation of tomato genes encoding light-signaling repressor proteins such as COP1, CUL4, DDB1, and DET1 (Fig. 3D), which are negative regulators of plastid division and maturation in tomato and other species (Chory and Peto, 1990; Kolotilin *et al.*, 2007; Wang *et al.*, 2008; Azari *et al.*, 2010*b*).

Collectively, these data suggest that fruit-localized PHYA positively influences tomato plastid division machinery via changes in the transcript abundance of both light- and cytokinin-signaling genes, whereas PHYB2 negatively regulates chlorophyll accumulation by controlling the expression of the master transcription factor of chloroplast development and maintenance, SlGLK2.

### Fruit-localized *PHYs* regulate starch metabolism during early fruit development

**Fig. 4.**
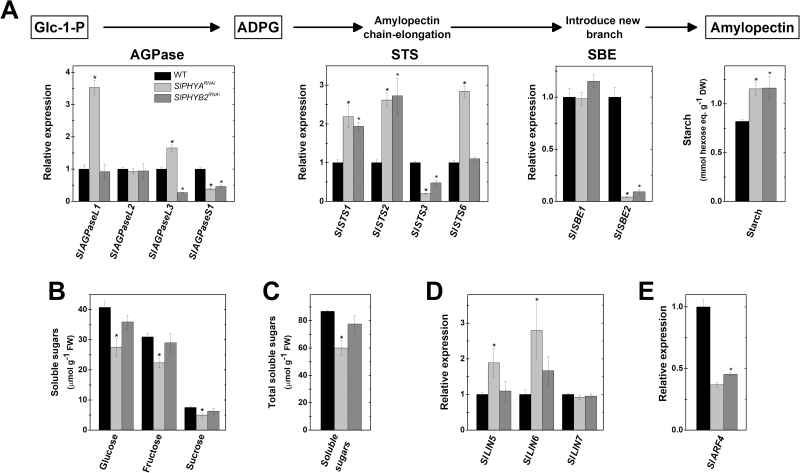
Fruit-localized phytochromes regulate sugar metabolism during early fruit development. (A) Schematic representation of the major steps of starch biosynthesis and graphs showing starch content and transcript abundance of starch biosynthesis-related genes in immature fruits. (B) Soluble sugar contents in immature fruits. (C) Summed values of the three soluble sugars analysed (i.e. sucrose + glucose + fructose). (D) Relative mRNA levels of tomato genes encoding invertases (*SlLIN*) in immature fruits. (E) Relative mRNA levels of *AUXIN RESPONSE FACTOR 4* (*SlARF4*) in immature fruits. For simplicity, the mean of the three values for the transgenic lines is shown. Values for each transgenic line are presented in Supplementary Figs S5, S6. Data are means (±SE) of at least three biological replicates. Statistically significant differences compared with the wild-type (WT) sample were determined using Student’s *t*-test: *P<0.05. IG, immature green; MG, mature green; Bk, breaker; RR, red ripe; Glc-1-P, glucose 1-phosphate; ADPG, adenosine diphosphate glucose; AGPase, ADP-glucose pyrophosphorylase; STS, starch synthase; SBE, starch branching enzyme.

Fruit-specific *SlPHYA* and *SlPHYB2* knockdown promoted starch accumulation during early fruit development (Fig. 4A). In both the WT and transgenic lines, the highest starch content was observed in immature green (IG) fruits, followed by slightly more reduced levels at the mature green (MG) stage, and undetectable levels from the breaker (Bk) stage onwards (Supplementary Fig. S5).

Compared to the WT genotype, marked differences in the transcript profiles of starch biosynthesis genes were observed in both *SlPHYA*- and *SlPHYB2*-silenced fruits (Fig. 4A, Supplementary Fig. S6). Catalysing the first committed step in starch biosynthesis, ADP-glucose pyrophosphorylase (AGPase) is a heterotetramer comprising a pair of small/catalytic and a pair of large/regulatory subunits (Kim *et al.*, 2007; Figueroa *et al.*, 2013). Among the tomato genes encoding the large AGPase subunits, both *SlAGPaseL1* and *SlAGPaseL3* were up-regulated whereas *SlAGPaseL2* mRNA levels remained unchanged in immature fruits of *SlPHYA*^*RNAi*^ plants. It is worth mentioning that *SlAGPaseL1* was the large AGPase subunit most expressed in immature tomato fruits (Supplementary Table S3; Petreikov *et al.*, 2006); therefore, the 3-fold increment in its mRNA levels correlates well with the higher starch levels and reduced soluble sugar levels detected in *SlPHYA*^*RNAi*^ immature fruits compared to the WT counterparts (Fig. 4, Supplementary Figs S5, S6).

*SlAGPaseS1*, which encodes the small/catalytic AGPase subunit, was consistently down-regulated throughout fruit development and ripening in both the *SlPHYA*^*RNAi*^ and *SlPHYB2*^*RNAi*^ lines. However, despite the negative impact of either *SlPHYA*- or *SlPHYB2*-silencing on *SlAGPaseS1* expression, this gene exhibited higher expression levels than those encoding AGPase large subunits (Supplementary Table S3), suggesting that the catalytic AGPase subunit was not limiting for starch biosynthesis in tomato fruits.

In both *SlPHYA*^*RNAi*^ and *SlPHYB2*^*RNAi*^ immature fruits, the starch synthase (STS)-encoding genes *SlSTS1* and *SlSTS2* were markedly up-regulated compared to WT fruits, whereas *SlSTS3* was slightly down-regulated. For *SlSTS6*, higher transcript accumulation was observed in *SlPHYA*^*RNAi*^ than in the WT throughout fruit development and ripening (i.e. IG to RR stage) (Supplementary Fig. S6). Finally, distinct expression patterns were observed for the starch branching enzyme (SBE)-encoding genes, as *SlSBE1* was up-regulated in all the transgenic lines from MG to Bk stage whereas *SlSBE2* was down-regulated in both *SlPHYA*^*RNAi*^ and *SlPHYB2*^*RNAi*^ from IG to RR stage (Supplementary Fig. S6).

The increased accumulation of starch in *SlPHYA*^*RNAi*^ fruits correlated well with higher mRNA levels of *SlLIN5* and *SlLIN6* (Fig. 4D), which encode cell-wall invertases critically important for sink activity in tomato (Fridman and Zamir, 2003; Kocal *et al.*, 2008). By applying an unsupervised method (i.e. principal component analysis, PCA) to search for patterns in the expression profiles of genes related to sink- and starch-biosynthesis, we demonstrated a clear separation of the WT, *SlPHYA*^*RNAi*^, and *SlPHYB2*^*RNAi*^ groups (Supplementary Fig. S7).

Previous findings have indicated that AUXIN RESPONSE FACTOR4 (SlARF4) is a major negative regulator of starch biosynthesis in early developing tomato fruits (Sagar *et al.*, 2013; Bianchetti *et al.*, 2017). Recent evidence also indicates that SlARF4 plays a repressor role in controlling the transcript abundance of sink-related genes, including *SlLIN5* and *SlLIN6* (Bianchetti *et al.*, 2017). In accordance with this, fruit-specific *SlPHYA* and *SlPHYB2* knockdown drastically reduced *SlARF4* mRNA abundance in early developing tomato fruits (Fig. 4E). Although the direct transcriptional regulation of tomato *AGPase*, *STS*, and *SBE* genes by transcription factors associated with auxin- or light-signaling remains to be determined, the presence of PBE-box, G-box, CA-hybrid, and/or CG-hybrid motifs (Martínez-García *et al.*, 2000; Song *et al.*, 2008) as well as canonical and/or degenerated ARF-binding Auxin Response Element (AuxRE) motifs within the 3-kb promoter sequence of these genes (Supplementary Fig. S8) is consistent with the hypothesis that light- and/or auxin-related transcription factors might directly control the expression of starch biosynthesis-related genes. Similarly, PIF, HY5, and/or ARF-binding motifs have also been identified within the promoter sequences of *SlLIN5* and *SlLIN6* genes (Bianchetti *et al.*, 2017).

### PHY-dependent regulation of fruit carotenoid biosynthesis is associated with transcriptional changes in light- and auxin-signaling genes

**Fig. 5.**
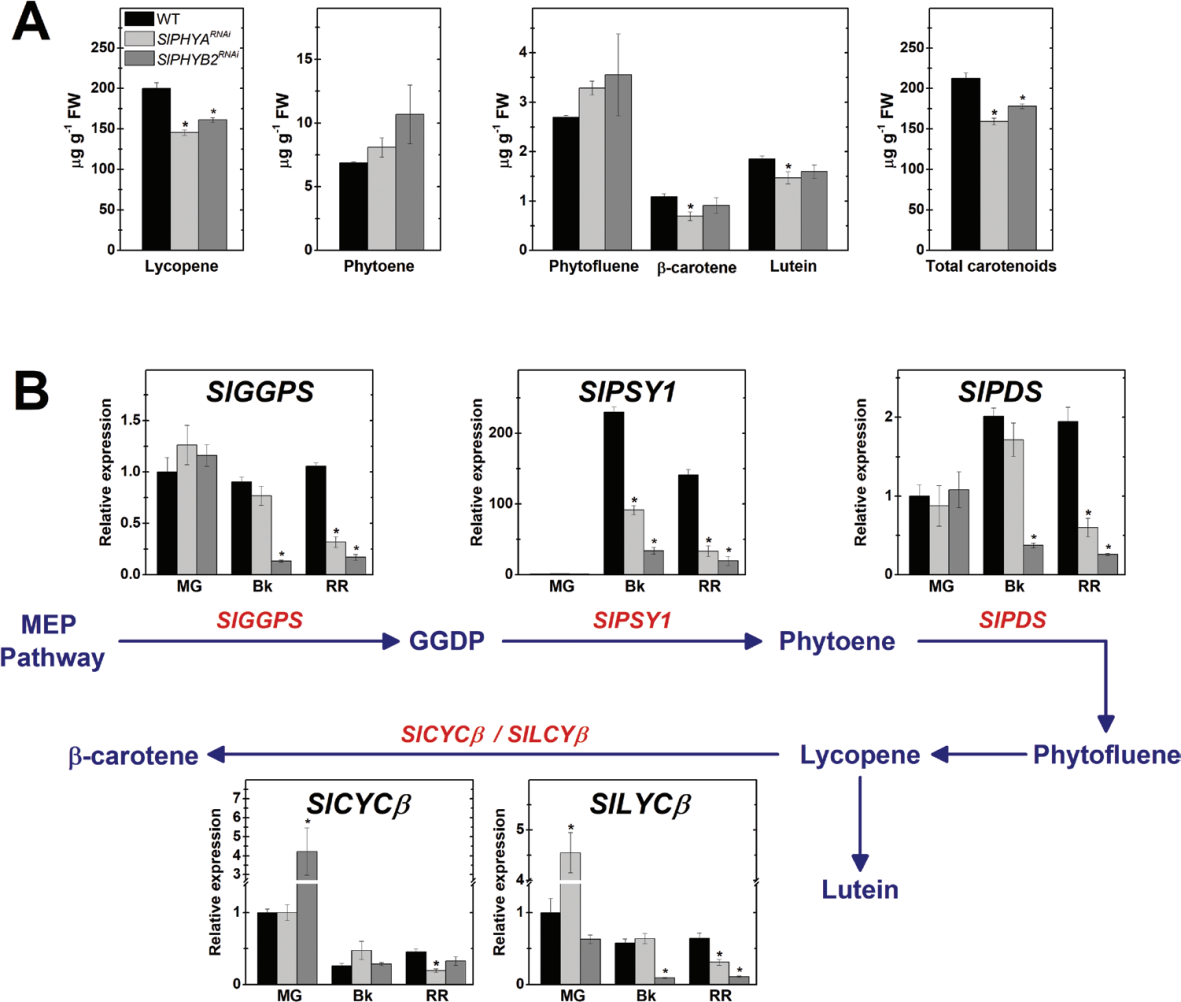
Fruit-specific *SlPHYA* or *SlPHYB2* knockdown represses carotenoid biosynthesis during tomato fruit ripening. (A) Lycopene, phytoene, phytofluene, β-carotene, lutein, and total carotenoid content in red ripe fruits. (B) Schematic representation of carotenoid biosynthetic pathway and graphs showing the transcript abundance of carotenoid biosynthesis genes in ripening fruits. Intermediate reactions are omitted. For simplicity, the mean of the three values for the transgenic lines is shown. Values for each transgenic line are presented in Supplementary Fig. S9, Supplementary Table S4. Data are means (±SE) of at least three biological replicates. Statistically significant differences compared with the wild-type (WT) sample were determined using Student’s *t*-test: *P<0.05. MG, mature green; Bk, breaker; RR, red ripe; MEP, methylerythritol 4-phosphate; GGDP, geranylgeranyl diphosphate; GGPS, GGDP synthase; PSY, phytoene synthase; PDS, phytoene desaturase; LCYβ, chloroplast-specific β-lycopene cyclase; CYCβ, chromoplast-specific β-lycopene cyclase. (This figure is available in color at *JXB* online.)

The very well-characterized PHY-mediated signaling networks controlling carotenogenesis in vegetative tissues (Toledo-Ortiz *et al.*, 2010) contrasts with the considerably more limited information regarding the fruit-localized PHY-dependent signaling cascades regulating carotenoid biosynthesis in fleshy fruits (Llorente *et al.*, 2016*b*, 2017). Carotenoid profiling revealed a significant reduction in lycopene content in red ripe (RR) fruits of both the *SlPHYA*^*RNAi*^ and *SlPHYB2*^*RNAi*^ lines compared to the WT (Fig. 5A, Supplementary Table S4). In contrast, the content of all other carotenoids analysed (i.e. phytoene, phytofluene, β-carotene, and lutein) remained virtually unchanged in ripe fruits of the transgenic lines compared to WT counterparts. As lycopene is the main carotenoid accumulated in ripe tomato, fruit-specific *SlPHYA*- or *SlPHYB2*-knockdown led to a slight, yet significant, reduction in total carotenoid content compared to the WT genotype (Fig. 5A, Supplementary Table S4). In accordance with this, significantly lower mRNA levels of genes encoding carotenoid biosynthesis-related enzymes such as GERANYLGERANYL DIPHOSPHATE SYNTHASE (GGPS), PHYTOENE SYNTHASE 1 (PSY1), and PHYTOENE DESATURASE (PDS) were observed in ripe fruits of *SlPHYA* and *SlPHYB2*-silenced lines than in WT counterparts (Fig. 5B, Supplementary Fig. S9). In line with the relatively limited reduction in total carotenoids, no significant differences in lipophilic antioxidant activity were observed between ripe WT and transgenic fruits (Supplementary Table S5). Interestingly, however, red ripe *SlPHYB2*-down-regulated fruits exhibited increased hydrophilic antioxidant activity compared to the WT, which may be associated with the higher content of total phenolics also detected in *SlPHYB2*^*RNAi*^ ripe fruits (Supplementary Table S5).

**Fig. 6.**
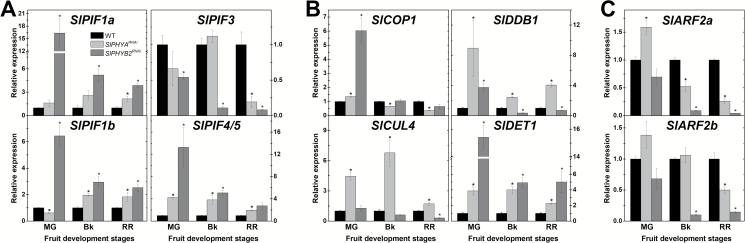
PHY-dependent regulation of fruit carotenogenesis is associated with transcriptional changes in auxin- and light-signaling genes. (A) Transcript abundance of tomato genes encoding PHYTOCHROME INTERACTING FACTORs (SlPIFs). (B) Transcript abundance of tomato genes encoding the light-signaling repressors CONSTITUTIVE PHOTOMORPHOGENIC 1 (SlCOP1), CULLIN 4 (SlCUL4), UV-DAMAGED DNA BINDING PROTEIN 1 (SlDDB1), and DE-ETIOLATED1 (SlDET1). (C) Transcript abundance of the tomato *AUXIN RESPONSIVE FACTOR 2a* and *2b* (*SlARF2a* and *SlARF2b*) genes. For simplicity, the mean of the three values for the transgenic lines is shown. Values for each transgenic line are presented in Supplementary Fig. S10. Data are means (±SE) of at least three biological replicates. Statistically significant differences compared with the wild-type (WT) sample were determined using Student’s *t*-test: *P<0.05. MG, mature green; Bk, breaker; RR, red ripe.

Accumulating evidence indicates that light-signaling repressors such as SlPIF1a, SlCOP1, SlCUL4, SlDDB1, and SlDET1 negatively regulate carotenoid biosynthesis in tomato fruits (Azari *et al.*, 2010*b*; Llorente *et al.*, 2016*b*) whereas auxin response factors such as SlARF2a and SlARF2b play the opposite role (Hao *et al.*, 2015). To gain insight into the potential role played by these signaling components during the PHY-dependent regulation of carotenoid biosynthesis in tomato fruits, the transcript abundance of their encoding genes was profiled in both *SlPHYA*^*RNAi*^ and *SlPHYB2*^*RNAi*^ ripening fruits (Fig. 6, Supplementary Fig. S10). Among the four *SlPIF* genes most highly expressed in fruits (Rosado *et al.*, 2016), *SlPIF1a*, *SlPIF1b*, and *SlPIF4/5* mRNA levels were significantly higher in *SlPHYB2*-down-regulated fruits compared to the WT counterparts during fruit ripening (MG, Bk, and RR stages), whereas the opposite was observed for *SlPIF3* transcripts. Although less pronounced, the overall impacts of fruit-specific *SlPHYA* knockdown on tomato *PIF* expression profiles were similar to those observed in the *SlPHYB2*^*RNAi*^ lines (Fig. 6, Supplementary Fig. S10).

Among the genes encoding light-signaling repressors, *SlCUL4*, *SlDDB1*, and *SlDET1* exhibited significantly higher mRNA levels in *SlPHYA*-silenced fruits in comparison to the WT at all fruit development stages analysed (Fig. 6B, Supplementary Fig. S10). Moreover, strikingly higher *SlDET1* transcript abundance was also detected in *SlPHYB2*-knockdown compared to WT fruits at all ripening stages (i.e. MG, Bk, and RR) whereas *SlCOP1* and *SlDDB1* mRNA levels were also up-regulated in *SlPHYB2*^*RNAi*^ fruits exclusively at the MG stage. Transcript levels of the positive regulators of tomato fruit carotenogenesis *SlARF2a* and *SlARF2b* were considerably lower in *SlPHYA*^*RNAi*^ and *SlPHYB2*^*RNAi*^ fruits, particularly at the Bk and RR stages (Fig. 6C, Supplementary Fig. S10). A PCA plot in which the expression profile of carotenoid biosynthesis-related genes as well as *SlPIF*s, *SlCOP1*, *SlCUL4*, *SlDDB1*, *SlDET1*, *SlARF2a*, and *SlARF2b* were represented revealed that the WT, *SlPHYA*^*RNAi*^, and *SlPHYB2*^*RNAi*^ groups clearly separated from each other at the red ripe stage (Supplementary Fig. S11).

Altogether, these data suggest that both *SlPHYA* and *SlPHYB2* play overlapping roles in promoting the paralogues *SlARF2a* and *SlARF2b* and repressing light-signaling repressors such as *SlPIF1a*, *SlPIF1b*, *SlPIF4/5, SlCOP1*, *SlCUL4*, *SlDDB1*, and *SlDET1*, which in turn mediate the PHY-dependent regulation of carotenoid biosynthesis in ripening tomato fruits.

## Discussion

Studies performed on PHY-deficient mutants have suggested that PHY-dependent light perception participates in the regulation of several aspects of tomato fruit biology (Gupta *et al.*, 2014; Bianchetti *et al.*, 2017). Here, we applied a RNAi-mediated organ-specific silencing approach to investigate the impact of fruit-localized SlPHYs on tomato fruit physiology and quality traits. Differently from the pleiotropic phenotypical alterations observed in *phy* mutants (Gupta *et al.*, 2014; Bianchetti *et al.*, 2017), the fruit-specific silencing of the target *SlPHY* genes resulted in no obvious impacts on plant vegetative growth and overall yield. This suggests that the perturbation in fruit metabolism caused by the fruit-specific SlPHY manipulation does not propagate from fruits to the rest of the plant, which agrees with the limited transference of substances out of this predominantly sink organ.

In a previous work, we demonstrated that a global deficiency in functional PHYs drastically reduces chlorophyll content and chloroplast abundance in tomato fruits (Bianchetti *et al.*, 2017). Therefore, the PHY-mediated regulation of plastid biogenesis and maturation widely reported for leaf tissues (Stephenson *et al.*, 2009; Oh and Montgomery, 2014; Melo *et al.*, 2016) seems to be conserved early in the development of tomato fruits. In this current work, it is further demonstrated that fruit-localized SlPHYA and SlPHYB2 play distinct roles in controlling chloroplast biogenesis and activity during early stages of tomato fruit development.

The results indicate that SlPHYA-mediated light perception promotes fruit chloroplast biogenesis and differentiation, as inferred from the reduced chlorophyll content, lower chloroplast abundance, and poorly-developed grana stacking detected in *SlPHYA*^*RNAi*^ immature fruits (Fig. 2). In line with this observation, an analysis of single and multiple *phy* mutants also suggested that SlPHYA is a major regulator of chlorophyll accumulation in tomato fruits (Gupta *et al.*, 2014). In land plants, chloroplast division depends on nucleus-encoded proteins that form ring structures at the division site (Jarvis and López-Juez, 2013). Our findings clearly demonstrate that fruit-localized *SlPHYA* influences the transcript levels of genes derived from the ancestral prokaryotic cell-division machinery, such as *SlFtsZ* (i.e. *SlFtsZ1*, *SlFtsZ2*) and *SlARC*s (i.e. *SlARC3* and *SlARC6*), as well as those encoding chloroplast division-related proteins specific to land plants, such as *SlPDV2*. In Arabidopsis, PDV2 determines the rate of chloroplast division and is positively regulated by cytokinins, being strongly promoted in transgenic plants overexpressing the cytokinin signaling-related transcription factor CRF2 (Okazaki *et al.*, 2009; Cortleven and Schmülling, 2015). *SlCRF2*, along with other *SlCRF* and *TRR* genes, were drastically repressed in *PHYA*-down-regulated fruits, implying that changes in cytokinin signaling mediate the PHYA-dependent regulation of plastid division during early stages of tomato fruit development. In agreement with this, accumulating evidence indicates that there is an intensive crosstalk between the PHY and cytokinin signaling cascades, with particular involvement of CRF and type-A ARR proteins (Salomé *et al.*, 2006; Oh *et al.*, 2009).

Fruit-specific *SlPHYA*-silencing also promoted the mRNA accumulation of genes encoding all the major light-signaling repressor proteins already described to negatively regulate chloroplast biogenesis in tomato fruits, i.e. *SlCOP1*, *SlCUL4*, *SlDDB1*, and *SlDET1* (Liu *et al.*, 2004; Kolotilin *et al.*, 2007; Wang *et al.*, 2008; Azari *et al.*, 2010*a*). Defective mutants or transgenic lines with reduced levels of each of these genes are known to develop more chloroplasts containing more grana/thylakoids in both leaves and immature fruits (Cookson *et al.*, 2003; Liu *et al.*, 2004; Kolotilin *et al.*, 2007; Wang *et al.*, 2008; Azari *et al.*, 2010*a*), which in some cases, such as in the *SlDET1*-knockout mutant, is associated with the up-regulation of plastid biogenesis-related genes (Kolotilin *et al.*, 2007). Therefore, the presence of fewer chloroplasts with poorly developed or almost no grana in immature fruits of the *SlPHYA*-suppressed lines agrees with the higher transcript abundance of *SlCOP1*, *SlDDB1*, and particularly *SlCUL4* and *SlDET1* in these transgenic lines compared to the WT genotype.

In contrast, fruit-localized SlPHYB2 was shown to play a negative role in chlorophyll accumulation, as evidenced by the increment in chlorophyll content in immature fruits of *SlPHYB2*^*RNAi*^ plants with no impact in chloroplast number in pericarp cells. As *SlPHYB2* fruit-specific silencing led to higher *SlGLK2* mRNA levels compared to the WT genotype, it seems plausible to suggest that the effect of SlPHYB2 on fruit chloroplasts is mediated by *SlGLK2*, the master regulator of chloroplast development in tomato fruits (Powell *et al.*, 2012). Further suggesting that the SlPHYB2-mediated regulation of *SlGLK2* expression is essential for the consequent changes in fruit chlorophyll accumulation, no obvious changes in chlorophyll content were observed in *phyb2* mutants from tomato varieties that lacked functional SlGLK2 proteins (Gupta *et al.*, 2014). In agreement with these findings, PHY-dependent transcriptional regulation of *GLK* genes has been increasingly reported in vegetative tissues of other plant species (Oh and Montgomery, 2014; Song *et al.*, 2014).

Alterations in chloroplast number, internal structure, and size during the early development of tomato fruits significantly impact the abundance of metabolites associated with organoleptic and nutritional quality at the ripe stage (Galpaz *et al.*, 2008; Cocaliadis *et al.*, 2014). Intense starch synthesis and degradation take place in tomato fruit chloroplasts at the unripe and breaker stages, respectively (Schaffer and Petreikov, 1997). Whereas the global deficiency in PHYs significantly reduces the starch content in immature tomato fruits (Bianchetti *et al.*, 2017), fruit-localized *SlPHYA* or *SlPHYB2* suppression increased fruit starch levels and markedly altered the transcriptional profile of starch biosynthesis-related genes at the immature green stage (Fig. 4). AGPase, which catalyses the rate-limiting reaction in the starch synthesis pathway, is both transcriptionally and post-translational regulated by light (Harn *et al.*, 2000; Geigenberger, 2011), although the role played by PHYs in this regulatory process remains elusive. During early fruit development, *SlPHYA*-suppressed fruits exhibited increased mRNA levels of both *SlAGPaseL1* and *SlAGPaseL3*, which encode AGPase large subunits, and *SlSTS1*, *SlSTS2*, and *SlSTS6*, which encode starch synthase enzymes, along with an increase in starch accumulation and reduced soluble sugar content, thus indicating a repressor role for fruit-localized SlPHYA on the first steps of starch synthesis in tomato fruits. Whether the up-regulation of starch biosinthesis-related genes is a compensatory mechanism to cope with the fewer and poorly developed chloroplasts observed in *SlPHYA*^*RNAi*^ immature fruits remains to be investigated. In contrast, the increased starch accumulation detected in *SlPHYB2*-silenced immature fruits was not associated with increments in transcript abundance of AGPase-encoding genes nor with prominent reductions in soluble sugars, but instead were accompanied by increments in *SlSTS1* and *SlSTS2* mRNA levels. Furthermore, as no significant alterations in plastid abundance or internal structure were observed in *SlPHYB2*^*RNAi*^ immature fruits, it seems likely that this genetic manipulation caused less prominent changes than *SlPHYA*-silencing on reactions taking place within fruit chloroplasts, including starch biosynthesis. Altogether, these findings suggest that SlPHYA and SlPHYB2 negatively regulate starch synthesis via overlapping, yet distinct, mechanisms.

The influence of auxin on fruit sugar metabolism has been increasingly reported (Purgatto *et al.*, 2002; Yuan and Carbaugh, 2007; Bianchetti *et al.*, 2017). In tomato, *SlARF4* has been described as a key negative regulator of starch synthesis during early fruit development via the transcriptional and post-transcriptional down-regulation of AGPase (Sagar *et al.*, 2013). Recent findings have also indicated that PHYs strictly regulate the transcript abundance of this particular auxin response factor in both vegetative (Melo *et al.*, 2016) and fruit tissues (Bianchetti *et al.*, 2017). In line with this, the increased starch accumulation in pre-ripening *SlPHYA*- and *SlPHYB2*-silenced fruits correlated well with the down-regulation of *SlARF4* in these transgenic lines (Fig. 4). In fact, *SlPHYA*^*RNAi*^ rather than *SlPHYB2*^*RNAi*^ exhibited the most expressive decrease in *SlARF4*, and only the former displayed increased mRNA levels of AGPase-encoding genes in immature fruits. Together, these data strongly suggest that fruit-localized PHYA, and to some extent SlPHYB2, positively modulates SlARF4, which in turn represses starch biosynthetic enzymes, such as AGPase and STS, consequently limiting starch synthesis in pre-ripening tomato fruits.

Previous findings indicated that a global deficiency in functional phytochromes transcriptionally represses both sink-related and starch biosynthesis-related enzymes in early developing tomato fruits, suggesting a promotive role of PHYs on the regulation of these processes (Bianchetti *et al.*, 2017). However, it remained unclear whether these responses were dependent on fruit-localized PHYs or were the consequence of collateral negative effects of the global PHY deficiency on vegetative plant growth. Here, we shed light on this topic by showing that fruit-localized SlPHYA, and to some extent SlPHYB2, repress both starch metabolism and key determinants of tomato fruit sink strength, including *SlLIN5* transcript accumulation (Fridman and Zamir, 2003; Kocal *et al.*, 2008). Consequently, the down-regulation in starch synthesis and sink activity previously observed in fruits of the PHY-deficient mutant *aurea* (Bianchetti *et al.*, 2017) may be due either to limitations in vegetative growth and metabolism or to the combinatory effect of the deficiency in all phytochromes instead of only in SlPHYA or SlPHYB2. Moreover, it also seems tempting to suggest that the fewer and poorly-developed chloroplasts detected in *SlPHYA*^*RNAi*^ immature fruits restrict photoassimilate production via fruit photosynthesis; therefore, the observed up-regulation of sink-related genes in transgenic fruits may represent a compensatory mechanism to maintain fruit growth and intense starch accumulation despite potential limitations in fruit-localized photoassimilation.

The link between PHY-dependent light perception and carotenoid metabolism in both vegetative and fruit tissues has been highlighted by a number of studies (Alba *et al.*, 2000*a*; Llorente *et al.*, 2016*b*). Exposure of wild-type tomato fruits to red light (Alba *et al.*, 2000*a*) or constitutively silencing of *SlPIF1a* (Llorente *et al.*, 2016*b*) promotes tomato fruit lycopene accumulation, thereby implying a positive role of PHY-dependent signaling cascades in the fruit carotenoid biosynthetic pathway. Consistent with this, our findings indicate that fruit-localized SlPHYA and SlPHYB2 positively influence the transcript accumulation of all the major carotenoid biosynthesis-related genes, including *SlGGPS*, *SlPSY1*, *SlPDS*, *SlCYCβ*, and *SlLYCβ*, consequently modifying the lycopene and total carotenoid content in this fleshy fruit. Light-signaling repressor proteins such as SlDET1, SlDDB1, SlCOP1, SlCUL4, and more recently SlPIF1a have been identified as key negative regulators of tomato fruit carotenoid synthesis (Liu *et al.*, 2004; Kolotilin *et al.*, 2007; Wang *et al.*, 2008; Azari *et al.*, 2010*a*; Llorente *et al.*, 2016*b*). Among these, the transcription factor SlPIF1a was shown to directly bind to the promoter of *SlPSY1* to repress fruit carotenogenesis (Llorente *et al.*, 2016*b*), thus resembling the action of its ortholog in Arabidopsis (AtPIF1) in controlling carotenoid biosynthesis in leaf tissues (Toledo-Ortiz *et al.*, 2010). Therefore, the marked up-regulation of *SlDET1*, *SlDDB1*, *SlCOP1*, *SlCUL4*, *SlPIF1a*, and *SlPIF1b* together with the overall repression of carotenoid biosynthesis observed in both *SlPHYA*- and *SlPHYB2*-silenced fruits imply that light-signaling repressor proteins participate in SlPHYA- and SlPHYB2-mediated regulation of fruit carotenogenesis.

In addition, it is becoming increasingly well established that auxin represses tomato ripening and down-regulates lycopene biosynthetic genes (Su *et al.*, 2015). Among tomato *ARF* genes, two paralogs, *SlARF2a* and *SlARF2b*, have emerged as key positive regulators of tomato fruit ripening and lycopene accumulation (Hao *et al.*, 2015). Either *SlPHYA* or *SlPHYB2* fruit-specific silencing profoundly reduced both *SlARF2a* and *SlARF2b*, suggesting the involvement of these auxin signaling elements in the PHY-dependent regulation of carotenoid biosynthesis in tomato fruits.

**Fig. 7.**
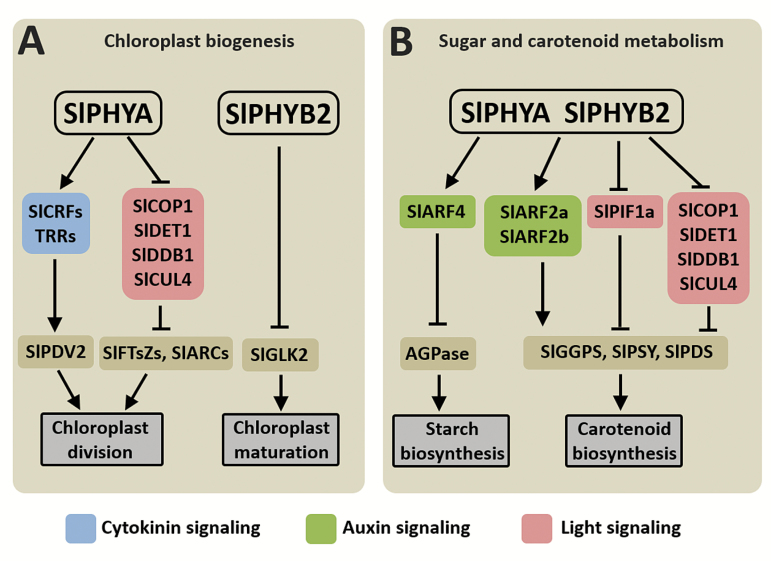
Proposed model for phytochrome-mediated signaling events controlling chloroplast biogenesis, and sugar and carotenoid metabolism in tomato fruits. (A) SlPHYA- and SlPHYB2-dependent light perception regulate fruit plastid division and maturation, respectively. By promoting key members of the cytokinin signaling-related *CRF* and *TRR* gene family, SlPHYA up-regulates SlPDV2, a rate-limiting component of the plastid division machinery. Moreover, the SlPHYA-mediated down-regulation of light-signaling repressors, such as SlCOP1, SlDET1, SlDDB1, and SlCUL4, induces other major components of the chloroplast division machinery, such as SlFTsZs and SlARCs. In contrast, Sl-PHYB2 represses the chloroplast differentiation transcription factor SlGLK2, consequently limiting chloroplast differentiation during early fruit development. (B) Fruit-localized SlPHYA and SlPHYB2 play overlapping roles in repressing and promoting starch and carotenoid biosynthesis, respectively. Both SlPHYA and SlPHYB2 induce SlARF4, a negative regulator of AGPase and starch accumulation in tomato fruits. In contrast, these same photoreceptors promote both *SlARF2* paralogues and inhibit all the major genes encoding light-signaling repressor proteins, consequently up-regulating most components of the tomato carotenoid biosynthetic route. Arrows at the ends of lines indicate stimulatory effects, whereas bars indicate inhibitory effects. AGPase, ADP-glucose pyrophosphorylase; ARC, accumulation and replication of chloroplasts; ARF, auxin response factor; COP1, constitutive photomorphogenic 1; CRF, cytokinin response factor; CUL4, cullin 4; DDB1, UV-damaged DNA binding protein 1; DET1, de-etiolated1; FtsZ, filamentous temperature sensitive-Z; GGPS, geranylgeranyl pyrophosphate synthase; GLK2, golden2-like-2; PDS, phytoene desaturase; PDV2, plastid division 2; PIF, phytochrome interacting factor; PSY, phytoene synthase; TRR, tomato response regulator.

Overall, our results shed light on the specific role played by fruit-localized phytochromes and their downstream signaling cascades, showing that plastid division, as well as sugar and carotenoid metabolism, are profoundly regulated by SlPHYA- and SlPHYB2-mediated light perception. A model summarizing the influence of fruit-localized SlPHYs on tomato fruit physiology is presented in Fig. 7. According to this model, SlPHYA and SlPHYB2 play overlapping roles in regulating starch and carotenoid biosynthesis, whereas they differentially regulate distinct aspects of fruit plastid biogenesis and maturation. Compared to SlPHYB2, SlPHYA-dependent light perception seems to play a major role in promoting plastid division and differentiation as well as in controlling sink-related transcripts in tomato fruits. The data implicate cytokinin signaling-related proteins as mediators of the SlPHYA-dependent regulation of the plastid division machinery, and specific *ARF* genes as potential intermediates in the PHY-mediated regulation of fruit sugar and carotenoid metabolism. Altogether, these findings show that fruit-specific manipulation of *PHY* genes represents a promising approach to differentially regulate multiple biosynthetic pathways and, consequently, to modify the nutritional value of edible fleshy fruits.

## Supplementary data

Supplementary data are available at *JXB* online.

Fig. S1. Transcriptional profile of tomato *PHY* genes in *PHY*-silenced fruits.

Fig. S2. Vegetative phenotypes of the transgenic plants.

Fig. S3. Visual phenotypes and color changes of *PHY*-silenced fruits.

Fig. S4. Plastid structure in *PHY*-silenced fruits.

Fig. S5. Carbohydrate profile in *PHY*-silenced fruits.

Fig. S6. Transcript abundance of starch biosynthetic genes in *PHY*-silenced fruits.

Fig. S7. PCA of the expression profile of sink-related and starch biosynthesis-related genes.

Fig. S8. HY5-, PIF-, and ARF-binding motifs identified in the promoter regions of starch biosynthesis-related tomato genes.

Fig. S9. Carotenoid metabolism during ripening in *PHY*-silenced fruits.

Fig. S10. Transcript abundance of photomorphogenesis- and auxin-related genes in *PHY*-silenced fruits.

Fig. S11. PCA of the expression profiles of photomorphogenesis-related, auxin-related, and carotenoid biosynthesis-related genes.

Table S1. Primer sequences.

Table S2. Homology of the RNAi fragments.

Table S3. Relative transcript ratios of *SlAGPase* in immature fruits.

Table S4. Carotenoid profiles in red ripe fruits.

Table S5. Antioxidant activity and total phenolics in red ripe fruits.

Supplementary Figures and Tables

## References

[CIT0001] Alba R, Cordonnier-Pratt MM, Pratt LH 2000a Fruit-localized phytochromes regulate lycopene accumulation independently of ethylene production in tomato. Plant Physiology 123, 363–370. 10806253 10.1104/pp.123.1.363 PMC59010

[CIT0002] Alba R, Kelmenson PM, Cordonnier-Pratt MM, Pratt LH 2000b The phytochrome gene family in tomato and the rapid differential evolution of this family in angiosperms. Molecular Biology and Evolution 17, 362–373. 10723737 10.1093/oxfordjournals.molbev.a026316

[CIT0003] Azari R, Reuveni M, Evenor D, Nahon S, Shlomo H, Chen L, Levin I 2010a Overexpression of *UV-DAMAGED DNA BINDING PROTEIN 1* links plant development and phytonutrient accumulation in high pigment-1 tomato. Journal of Experimental Botany 61, 3627–3637. 20566564 10.1093/jxb/erq176 PMC2921201

[CIT0004] Azari R, Tadmor Y, Meir A, Reuveni M, Evenor D, Nahon S, Shlomo H, Chen L, Levin I 2010b Light signaling genes and their manipulation towards modulation of phytonutrient content in tomato fruits. Biotechnology Advances 28, 108–118. 19850117 10.1016/j.biotechadv.2009.10.003

[CIT0005] Bianchetti RE, Cruz AB, Oliveira BS, Demarco D, Purgatto E, Peres LEP, Rossi M, Freschi L 2017 Phytochromobilin deficiency impairs sugar metabolism through the regulation of cytokinin and auxin signaling in tomato fruits. Scientific Reports 7, 7822. 28798491 10.1038/s41598-017-08448-2 PMC5552807

[CIT0006] Carvalho RF, Campos ML, Pino LE, Crestana SL, Zsögön A, Lima JE, Benedito VA, Peres LE 2011 Convergence of developmental mutants into a single tomato model system: ‘Micro-Tom’ as an effective toolkit for plant development research. Plant Methods 7, 18. 21714900 10.1186/1746-4811-7-18 PMC3146949

[CIT0007] Chaabouni S, Jones B, Delalande C, Wang H, Li Z, Mila I, Frasse P, Latché A, Pech JC, Bouzayen M 2009 Sl-IAA3, a tomato Aux/IAA at the crossroads of auxin and ethylene signalling involved in differential growth. Journal of Experimental Botany 60, 1349–1362. 19213814 10.1093/jxb/erp009 PMC2657550

[CIT0008] Chory J, Peto CA 1990 Mutations in the *DET1* gene affect cell-type-specific expression of light-regulated genes and chloroplast development in *Arabidopsis*. Proceedings of the National Academy of Sciences, USA 87, 8776–8780. 10.1073/pnas.87.22.8776 PMC55042 2247447

[CIT0009] Chow CN, Zheng HQ, Wu NY, Chien CH, Huang HD, Lee TY, Chiang-Hsieh YF, Hou PF, Yang TY, Chang WC 2016 PlantPAN 2.0: an update of plant promoter analysis navigator for reconstructing transcriptional regulatory networks in plants. Nucleic Acids Research 44, D1154–D1160. 26476450 10.1093/nar/gkv1035 PMC4702776

[CIT0010] Cocaliadis MF, Fernández-Muñoz R, Pons C, Orzaez D, Granell A 2014 Increasing tomato fruit quality by enhancing fruit chloroplast function. A double-edged sword?Journal of Experimental Botany 65, 4589–4598. 24723405 10.1093/jxb/eru165

[CIT0011] Cookson PJ, Kiano JW, Shipton CA, Fraser PD, Romer S, Schuch W, Bramley PM, Pyke KA 2003 Increases in cell elongation, plastid compartment size and phytoene synthase activity underlie the phenotype of the high pigment-1 mutant of tomato. Planta 217, 896–903. 12844264 10.1007/s00425-003-1065-9

[CIT0012] Cortleven A, Schmülling T 2015 Regulation of chloroplast development and function by cytokinin. Journal of Experimental Botany 66, 4999–5013. 25873684 10.1093/jxb/erv132

[CIT0013] Dai X, Zhao PX 2011 psRNATarget: a plant small RNA target analysis server. Nucleic Acids Research 39, W155–W159. 21622958 10.1093/nar/gkr319 PMC3125753

[CIT0014] Davuluri GR, van Tuinen A, Fraser PD, et al 2005 Fruit-specific RNAi-mediated suppression of *DET1* enhances carotenoid and flavonoid content in tomatoes. Nature Biotechnology 23, 890–895. 10.1038/nbt1108 PMC3855302 15951803

[CIT0015] Deng X-W, Quail PH 1992 Genetic and phenotypic characterization of *cop1* mutants of *Arabidopsis thaliana*. The Plant Journal 2, 83–95.

[CIT0016] Duek PD, Fankhauser C 2005 bHLH class transcription factors take centre stage in phytochrome signalling. Trends in Plant Science 10, 51–54. 15708340 10.1016/j.tplants.2004.12.005

[CIT0017] Enfissi EM, Barneche F, Ahmed I, et al 2010 Integrative transcript and metabolite analysis of nutritionally enhanced *DE-ETIOLATED1* downregulated tomato fruit. The Plant Cell 22, 1190–1215. 20435899 10.1105/tpc.110.073866 PMC2879742

[CIT0018] Expósito-Rodríguez M, Borges AA, Borges-Pérez A, Pérez JA 2008 Selection of internal control genes for quantitative real-time RT-PCR studies during tomato development process. BMC Plant Biology 8, 131. 19102748 10.1186/1471-2229-8-131 PMC2629474

[CIT0019] Fernandez AI, Viron N, Alhagdow M, et al 2009 Flexible tools for gene expression and silencing in tomato. Plant Physiology 151, 1729–1740. 19812183 10.1104/pp.109.147546 PMC2785966

[CIT0020] Figueroa CM, Kuhn ML, Falaschetti CA, Solamen L, Olsen KW, Ballicora MA, Iglesias AA 2013 Unraveling the activation mechanism of the potato tuber ADP-glucose pyrophosphorylase. PLoS ONE 8, e66824. 23826149 10.1371/journal.pone.0066824 PMC3691274

[CIT0021] Fridman E, Zamir D 2003 Functional divergence of a syntenic invertase gene family in tomato, potato, and Arabidopsis. Plant Physiology 131, 603–609. 12586884 10.1104/pp.014431 PMC166836

[CIT0022] Galpaz N, Wang Q, Menda N, Zamir D, Hirschberg J 2008 Abscisic acid deficiency in the tomato mutant *high-pigment 3* leading to increased plastid number and higher fruit lycopene content. The Plant Journal 53, 717–730. 17988221 10.1111/j.1365-313X.2007.03362.x

[CIT0023] Geigenberger P 2011 Regulation of starch biosynthesis in response to a fluctuating environment. Plant Physiology 155, 1566–1577. 21378102 10.1104/pp.110.170399 PMC3091114

[CIT0024] Giliberto L, Perrotta G, Pallara P, Weller JL, Fraser PD, Bramley PM, Fiore A, Tavazza M, Giuliano G 2005 Manipulation of the blue light photoreceptor cryptochrome 2 in tomato affects vegetative development, flowering time, and fruit antioxidant content. Plant Physiology 137, 199–208. 15618424 10.1104/pp.104.051987 PMC548851

[CIT0025] Giovannoni J, Nguyen C, Ampofo B, Zhong S, Fei Z 2017 The epigenome and transcriptional dynamics of fruit ripening. Annual Review of Plant Biology 68, 61–84. 10.1146/annurev-arplant-042916-040906 28226232

[CIT0026] Gupta SK, Sharma S, Santisree P, Kilambi HV, Appenroth K, Sreelakshmi Y, Sharma R 2014 Complex and shifting interactions of phytochromes regulate fruit development in tomato. Plant, Cell & Environment 37, 1688–1702. 10.1111/pce.12279 24433205

[CIT0027] Hao Y, Hu G, Breitel D, Liu M, Mila I, Frasse P, Fu Y, Aharoni A, Bouzayen M, Zouine M 2015 Auxin response factor SlARF2 is an essential component of the regulatory mechanism controlling fruit ripening in tomato. PLoS Genetics 11, e1005649. 26716451 10.1371/journal.pgen.1005649 PMC4696797

[CIT0028] Harn CH, Bae JM, Lee SS, Min SR, Liu JR 2000 Presence of multiple cDNAs encoding an isoform of ADP-glucose pyrophosphorylase large subunit from sweet potato and characterization of expression levels. Plant & Cell Physiology 41, 1235–1242. 11092908 10.1093/pcp/pcd049

[CIT0029] Hauser BA, Pratt LH, Cordonnier-Pratt MM 1997 Absolute quantification of five phytochrome transcripts in seedlings and mature plants of tomato (*Solanum lycopersicum* L.). Planta 201, 379–387. 9129340 10.1007/s004250050080

[CIT0030] Inagaki N, Kinoshita K, Kagawa T, Tanaka A, Ueno O, Shimada H, Takano M 2015 Phytochrome B mediates the regulation of chlorophyll biosynthesis through transcriptional regulation of ChlH and GUN4 in rice seedlings. PLoS ONE 10, e0135408. 26270815 10.1371/journal.pone.0135408 PMC4536196

[CIT0031] Jarvis P, López-Juez E 2013 Biogenesis and homeostasis of chloroplasts and other plastids. Nature Reviews Molecular Cell Biology 14, 787–802. 24263360 10.1038/nrm3702

[CIT0032] Kim D, Hwang SK, Okita TW 2007 Subunit interactions specify the allosteric regulatory properties of the potato tuber ADP-glucose pyrophosphorylase. Biochemical and Biophysical Research Communications 362, 301–306. 17707339 10.1016/j.bbrc.2007.07.162

[CIT0033] Kocal N, Sonnewald U, Sonnewald S 2008 Cell wall-bound invertase limits sucrose export and is involved in symptom development and inhibition of photosynthesis during compatible interaction between tomato and *Xanthomonas campestris* pv *vesicatoria*. Plant Physiology 148, 1523–1536. 18784281 10.1104/pp.108.127977 PMC2577280

[CIT0034] Kolotilin I, Koltai H, Tadmor Y, Bar-Or C, Reuveni M, Meir A, Nahon S, Shlomo H, Chen L, Levin I 2007 Transcriptional profiling of *high pigment-2dg* tomato mutant links early fruit plastid biogenesis with its overproduction of phytonutrients. Plant Physiology 145, 389–401. 17704236 10.1104/pp.107.102962 PMC2048735

[CIT0035] Kumar R, Khurana A, Sharma AK 2014 Role of plant hormones and their interplay in development and ripening of fleshy fruits. Journal of Experimental Botany 65, 4561–4575. 25028558 10.1093/jxb/eru277

[CIT0036] Lira BS, Gramegna G, Trench BA, et al 2017 Manipulation of a senescence-associated gene improves fleshy fruit yield. Plant Physiology 175, 77–91. 28710129 10.1104/pp.17.00452 PMC5580748

[CIT0037] Lira BS, Rosado D, Almeida J, de Souza AP, Buckeridge MS, Purgatto E, Guyer L, Hörtensteiner S, Freschi L, Rossi M 2016 Pheophytinase knockdown impacts carbon metabolism and nutraceutical content under normal growth conditions in tomato. Plant & Cell Physiology 57, 642–653. 26880818 10.1093/pcp/pcw021

[CIT0038] Liu YS, Roof S, Ye ZB, Barry C, van Tuinen A, Vrebalov J, Bowler C, Giovannoni J 2004 Manipulation of light signal transduction as a means of modifying fruit nutritional quality in tomato. Proceedings of the National Academy of Sciences, USA 101, 9897–9902. 10.1073/pnas.0400935101 PMC470770 15178762

[CIT0039] Llorente B, D’Andrea L, Rodríguez-Concepción M 2016a Evolutionary recycling of light signaling components in fleshy fruits: new insights on the role of pigments to monitor ripening. Frontiers in Plant Science 7, 263. 27014289 10.3389/fpls.2016.00263 PMC4780243

[CIT0040] Llorente B, D’Andrea L, Ruiz-Sola MA, Botterweg E, Pulido P, Andilla J, Loza-Alvarez P, Rodriguez-Concepcion M 2016b Tomato fruit carotenoid biosynthesis is adjusted to actual ripening progression by a light-dependent mechanism. The Plant Journal 85, 107–119. 26648446 10.1111/tpj.13094

[CIT0041] Llorente B, Martinez-Garcia JF, Stange C, Rodriguez-Concepcion M 2017 Illuminating colors: regulation of carotenoid biosynthesis and accumulation by light. Current Opinion in Plant Biology 37, 49–55. 28411584 10.1016/j.pbi.2017.03.011

[CIT0042] Martínez-García JF, Huq E, Quail PH 2000 Direct targeting of light signals to a promoter element-bound transcription factor. Science 288, 859–863. 10797009 10.1126/science.288.5467.859

[CIT0043] Melo NK, Bianchetti RE, Lira BS, Oliveira PM, Zuccarelli R, Dias DL, Demarco D, Peres LE, Rossi M, Freschi L 2016 Nitric oxide, ethylene, and auxin cross talk mediates greening and plastid development in deetiolating tomato seedlings. Plant Physiology 170, 2278–2294. 26829981 10.1104/pp.16.00023 PMC4825133

[CIT0044] Oh E, Kang H, Yamaguchi S, Park J, Lee D, Kamiya Y, Choi G 2009 Genome-wide analysis of genes targeted by PHYTOCHROME INTERACTING FACTOR 3-LIKE5 during seed germination in Arabidopsis. The Plant Cell 21, 403–419. 19244139 10.1105/tpc.108.064691 PMC2660632

[CIT0045] Oh S, Montgomery BL 2014 Phytochrome-dependent coordinate control of distinct aspects of nuclear and plastid gene expression during anterograde signaling and photomorphogenesis. Frontiers in Plant Science 5, 171. 24817873 10.3389/fpls.2014.00171 PMC4012200

[CIT0046] Okazaki K, Kabeya Y, Suzuki K, Mori T, Ichikawa T, Matsui M, Nakanishi H, Miyagishima SY 2009 The PLASTID DIVISION1 and 2 components of the chloroplast division machinery determine the rate of chloroplast division in land plant cell differentiation. The Plant Cell 21, 1769–1780. 19567705 10.1105/tpc.109.067785 PMC2714929

[CIT0047] Pepper A, Delaney T, Washburn T, Poole D, Chory J 1994 DET1, a negative regulator of light-mediated development and gene expression in arabidopsis, encodes a novel nuclear-localized protein. Cell 78, 109–116. 8033202 10.1016/0092-8674(94)90577-0

[CIT0048] Petreikov M, Shen S, Yeselson Y, Levin I, Bar M, Schaffer AA 2006 Temporally extended gene expression of the ADP-Glc pyrophosphorylase large subunit (*AgpL1*) leads to increased enzyme activity in developing tomato fruit. Planta 224, 1465–1479. 16770584 10.1007/s00425-006-0316-y

[CIT0049] Pino LE, Lombardi-Crestana S, Azevedo MS, Scotton DC, Borgo L, Quecini V, Figueira A, Peres LE 2010 The *Rg1* allele as a valuable tool for genetic transformation of the tomato ‘Micro-Tom’ model system. Plant Methods 6, 23. 20929550 10.1186/1746-4811-6-23 PMC2958934

[CIT0050] Piringer AA, Heinze PH 1954 Effect of light on the formation of a pigment in the tomato fruit cuticle. Plant Physiology 29, 467–472. 16654698 10.1104/pp.29.5.467 PMC540560

[CIT0051] Porra RJ, Thompson WA, Kriedemann PE 1989 Determination of accurate extinction coefficients and simultaneous equations for assaying chlorophylls *a* and *b* extracted with four different solvents: verification of the concentration of chlorophyll standards by atomic absorption spectroscopy. Biochimica et Biophysica Acta 975, 384–394.

[CIT0052] Powell AL, Nguyen CV, Hill T, et al 2012 *Uniform ripening* encodes a *Golden 2-like* transcription factor regulating tomato fruit chloroplast development. Science 336, 1711–1715. 22745430 10.1126/science.1222218

[CIT0053] Purgatto E, Oliveira do Nascimento JR, Lajolo FM, Cordenunsi BR 2002 The onset of starch degradation during banana ripening is concomitant to changes in the content of free and conjugated forms of indole-3-acetic acid. Journal of Plant Physiology 159, 1105–1111.

[CIT0054] Quadrana L, Almeida J, Otaiza SN, et al 2013 Transcriptional regulation of tocopherol biosynthesis in tomato. Plant Molecular Biology 81, 309–325. 23247837 10.1007/s11103-012-0001-4

[CIT0055] Rosado D, Gramegna G, Cruz A, Lira BS, Freschi L, de Setta N, Rossi M 2016 Phytochrome interacting factors (PIFs) in *Solanum lycopersicum*: diversity, evolutionary history and expression profiling during different developmental processes. PLoS ONE 11, e0165929. 27802334 10.1371/journal.pone.0165929 PMC5089782

[CIT0056] Ruijter JM, Ramakers C, Hoogaars WMH, Karlen Y, Bakker O, van den Hoff MJB, Moorman AFM 2009 Amplification efficiency: linking baseline and bias in the analysis of quantitative PCR data. Nucleic Acids Research 37, e45. 19237396 10.1093/nar/gkp045 PMC2665230

[CIT0057] Sagar M, Chervin C, Mila I, et al 2013 SlARF4, an auxin response factor involved in the control of sugar metabolism during tomato fruit development. Plant Physiology 161, 1362–1374. 23341361 10.1104/pp.113.213843 PMC3585602

[CIT0058] Salomé PA, To JP, Kieber JJ, McClung CR 2006 Arabidopsis response regulators ARR3 and ARR4 play cytokinin-independent roles in the control of circadian period. The Plant Cell 18, 55–69. 16326927 10.1105/tpc.105.037994 PMC1323484

[CIT0059] Schaffer AA, Petreikov M 1997 Sucrose-to-starch metabolism in tomato fruit undergoing transient starch accumulation. Plant Physiology 113, 739–746. 12223639 10.1104/pp.113.3.739 PMC158191

[CIT0060] Schofield A, Paliyath G 2005 Modulation of carotenoid biosynthesis during tomato fruit ripening through phytochrome regulation of phytoene synthase activity. Plant Physiology and Biochemistry 43, 1052–1060. 16442806 10.1016/j.plaphy.2005.10.006

[CIT0061] Schrager-Lavelle A, Herrera LA, Maloof JN 2016 Tomato phyE is required for shade avoidance in the absence of phyB1 and phyB2. Frontiers in Plant Science 7, 1275. 27695458 10.3389/fpls.2016.01275 PMC5025638

[CIT0062] Schroeder DF, Gahrtz M, Maxwell BB, Cook RK, Kan JM, Alonso JM, Ecker JR, Chory J 2002 De-etiolated 1 and damaged DNA binding protein 1 interact to regulate Arabidopsis photomorphogenesis. Current Biology 12, 1462–1472. 12225661 10.1016/s0960-9822(02)01106-5

[CIT0063] Shi X, Gupta S, Rashotte AM 2012 *Solanum lycopersicum cytokinin response factor* (*SlCRF*) genes: characterization of CRF domain-containing ERF genes in tomato. Journal of Experimental Botany 63, 973–982. 22068146 10.1093/jxb/err325 PMC3254692

[CIT0064] Singleton VL, Rossi JA 1965 Colorimetry of total phenolics with phosphomolybdic-phosphotungstic acid reagents. American Journal of Enology and Viticulture 16, 144–158.

[CIT0065] Song Y, Yang C, Gao S, Zhang W, Li L, Kuai B 2014 Age-triggered and dark-induced leaf senescence require the bHLH transcription factors PIF3, 4, and 5. Molecular Plant 7, 1776–1787. 25296857 10.1093/mp/ssu109 PMC4261840

[CIT0066] Song YH, Yoo CM, Hong AP, et al 2008 DNA-binding study identifies C-box and hybrid C/G-box or C/A-box motifs as high-affinity binding sites for STF1 and LONG HYPOCOTYL5 proteins. Plant Physiology 146, 1862–1877. 18287490 10.1104/pp.107.113217 PMC2287355

[CIT0067] Stephenson PG, Fankhauser C, Terry MJ 2009 PIF3 is a repressor of chloroplast development. Proceedings of the National Academy of Sciences, USA 106, 7654–7659. 10.1073/pnas.0811684106 PMC2678601 19380736

[CIT0068] Su L, Diretto G, Purgatto E, Danoun S, Zouine M, Li Z, Roustan JP, Bouzayen M, Giuliano G, Chervin C 2015 Carotenoid accumulation during tomato fruit ripening is modulated by the auxin–ethylene balance. BMC Plant Biology 15, 114. 25953041 10.1186/s12870-015-0495-4 PMC4424491

[CIT0069] Suguiyama VF, Silva EA, Meirelles ST, Centeno DC, Braga MR 2014 Leaf metabolite profile of the Brazilian resurrection plant *Barbacenia purpurea* Hook. (Velloziaceae) shows two time-dependent responses during desiccation and recovering. Frontiers in Plant Science 5, 96. 24672534 10.3389/fpls.2014.00096 PMC3953666

[CIT0070] Thomann A, Dieterle M, Genschik P 2005 Plant CULLIN-based E3s: phytohormones come first. FEBS Letters 579, 3239–3245. 15943967 10.1016/j.febslet.2005.02.068

[CIT0071] Toledo-Ortiz G, Huq E, Rodríguez-Concepción M 2010 Direct regulation of phytoene synthase gene expression and carotenoid biosynthesis by phytochrome-interacting factors. Proceedings of the National Academy of Sciences, USA 107, 11626–11631. 10.1073/pnas.0914428107 PMC2895139 20534526

[CIT0072] **Tomato Genome Consortium** 2012 The tomato genome sequence provides insights into fleshy fruit evolution. Nature 485, 635–641. 22660326 10.1038/nature11119 PMC3378239

[CIT0073] van Tuinen A, Kerckhoffs LH, Nagatani A, Kendrick RE, Koornneef M 1995a Far-red light-insensitive, phytochrome A-deficient mutants of tomato. Molecular & General Genetics 246, 133–141. 7862083 10.1007/BF00294675

[CIT0074] van Tuinen A, Kerckhoffs L, Nagatani A, Kendrick RE, Koornneef M 1995b A temporarily red light-insensitive mutant of tomato lacks a light-stable, B-like phytochrome. Plant Physiology 108, 939–947. 12228517 10.1104/pp.108.3.939 PMC157443

[CIT0075] Wang S, Liu J, Feng Y, Niu X, Giovannoni J, Liu Y 2008 Altered plastid levels and potential for improved fruit nutrient content by downregulation of the tomato DDB1-interacting protein CUL4. The Plant Journal 55, 89–103. 18363785 10.1111/j.1365-313X.2008.03489.x

[CIT0076] Weller JL, Schreuder ME, Smith H, Koornneef M, Kendrick RE 2000 Physiological interactions of phytochromes A, B1 and B2 in the control of development in tomato. The Plant Journal 24, 345–356. 11069708 10.1046/j.1365-313x.2000.00879.x

[CIT0077] Xu P, Zhang Y, Kang L, Roossinck MJ, Mysore KS 2006 Computational estimation and experimental verification of off-target silencing during posttranscriptional gene silencing in plants. Plant Physiology 142, 429–440. 16920874 10.1104/pp.106.083295 PMC1586062

[CIT0078] Yuan R, Carbaugh DH 2007 Effects of NAA, AVG, and 1-MCP on ethylene biosynthesis, preharvest fruit drop, fruit maturity, and quality of ‘Golden Supreme’ and ‘Golden Delicious’ apples. HortScience 42, 101–105.

