# Supplementary material for: Fruit-localized phytochromes regulate plastid biogenesis, starch synthesis, and carotenoid metabolism in tomato
Source: J Exp Bot. 2018 Apr 18;69(15):3573–86. doi: 10.1093/jxb/ery145 (PMC6022544; doi:10.1093/jxb/ery145)
Supplement: Supplementary Figures and Tables [file ery145_suppl_supplementary_figures_tables.pdf]

Fruit-localized phytochromes regulate plastid biogenesis, starch synthesis and carotenoid metabolism in tomato

Authors: Ricardo Ernesto Bianchetti, Bruno Silvestre Lira, Scarlet Santos Monteiro, Diego Demarco, Eduardo Purgatto, Christophe Rothan, Magdalena Rossi, Luciano Freschi

The following Supplementary data is available for this article:

Fig. S1. Transcriptional profile of tomato *PHY* genes in *PHY*-silenced fruits.

Fig. S2. Vegetative phenotypes of the transgenic plants.

Fig. S3. Visual phenotypes and color change of *PHY*-silenced fruits.

Fig. S4. Plastid structure in *PHY*-silenced fruits.

Fig. S5. Carbohydrate profile in *PHY*-silenced fruits.

Fig. S6. Transcript abundance of starch biosynthetic genes in *PHY*-silenced fruits.

Fig. S7. PCA of the expression profile of sink-related and starch biosynthesis-related genes.

Fig. S8. HY5-, PIF- and ARF-binding motifs identified in the promoter region of starch biosynthesis-related tomato genes.

Fig. S9. Carotenoid metabolism during ripening in *PHY*-silenced fruits.

Fig. S10. Transcript abundance of photomorphogenesis- and auxin-related genes in *PHY*-silenced fruits.

Fig. S11. PCA of the expression profile of photomorphogenesis-related, auxin-related and carotenoid biosynthesis-related genes.

Table S1. Primer sequences.

Table S2. Homology of the RNAi fragments.

Table S3. Relative transcript ratios of *SlAGPase* in immature fruits.

Table S4. Carotenoid profile in red ripe fruits.

Table S5. Antioxidant activity and total phenolics in red ripe fruits.

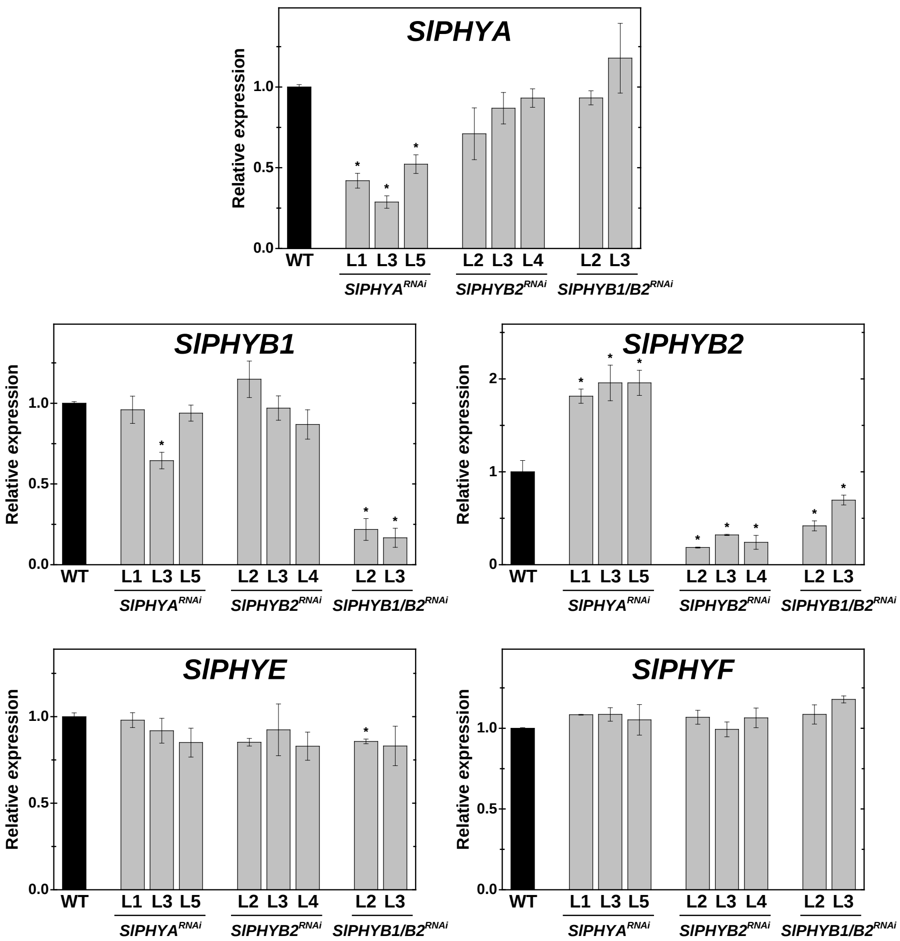

Figure S1. Transcriptional profile of tomato *PHY* genes in *PHY*-silenced fruits. Relative mRNA levels of *SlPHYA*, *SlPHYB1*, *SlPHYB2, SlPHYE* and *SlPHYF* in mature green fruits of *SlPHYA*^{RNAi}, *SlPHYB2*^{RNAi} and *SlPHYB1/B2*^{RNAi} lines. Data shown are mean ± SE of at least three biological replicates normalized against the wildtype (WT) sample. Asterisks indicate statistically significant differences (Student's t-test, $P<0.05$) compared with the WT sample.

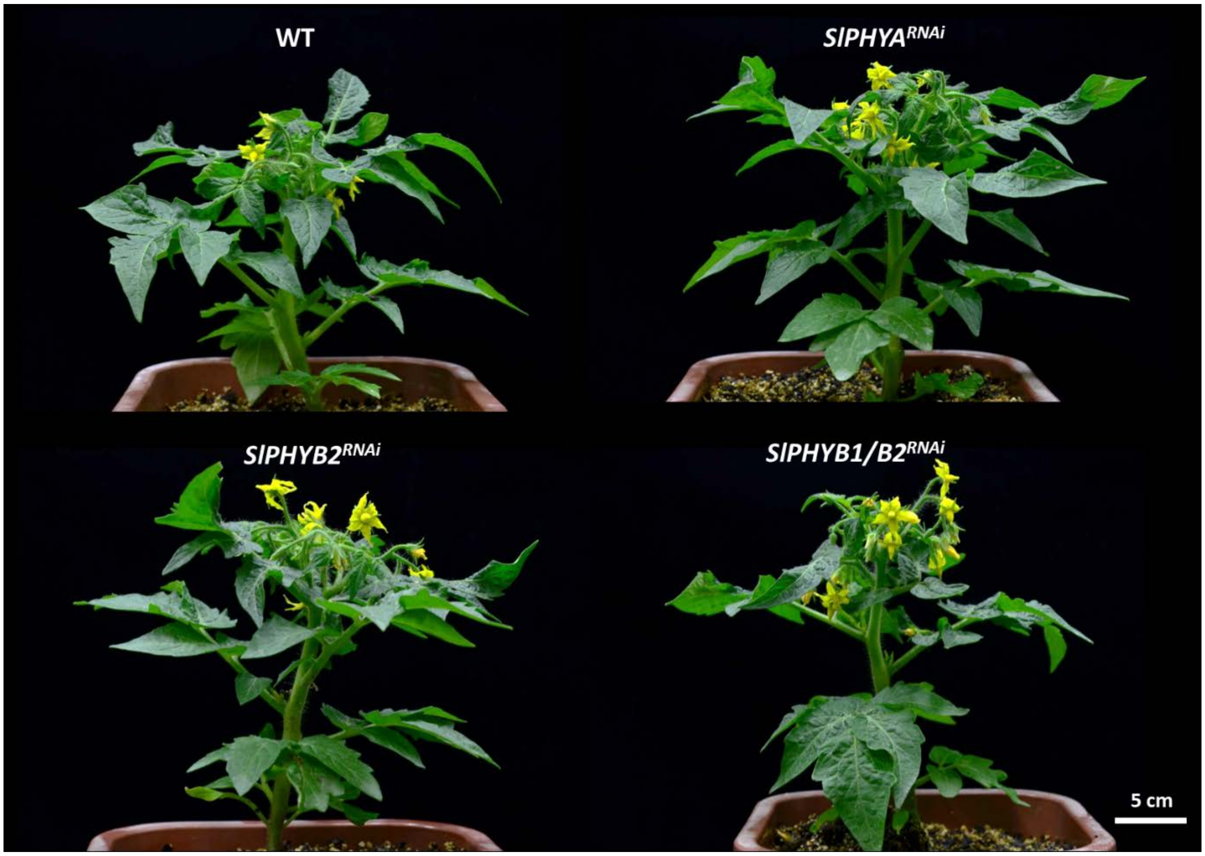

Figure S2. Vegetative phenotypes of the transgenic plants. Phenotypes of *SlPHYA*^{RNAi}, *SlPHYB2*^{RNAi}, *SlPHYB1/B2*^{RNAi} and wildtype (WT) plants at the flowering stage (42-day-old plants).

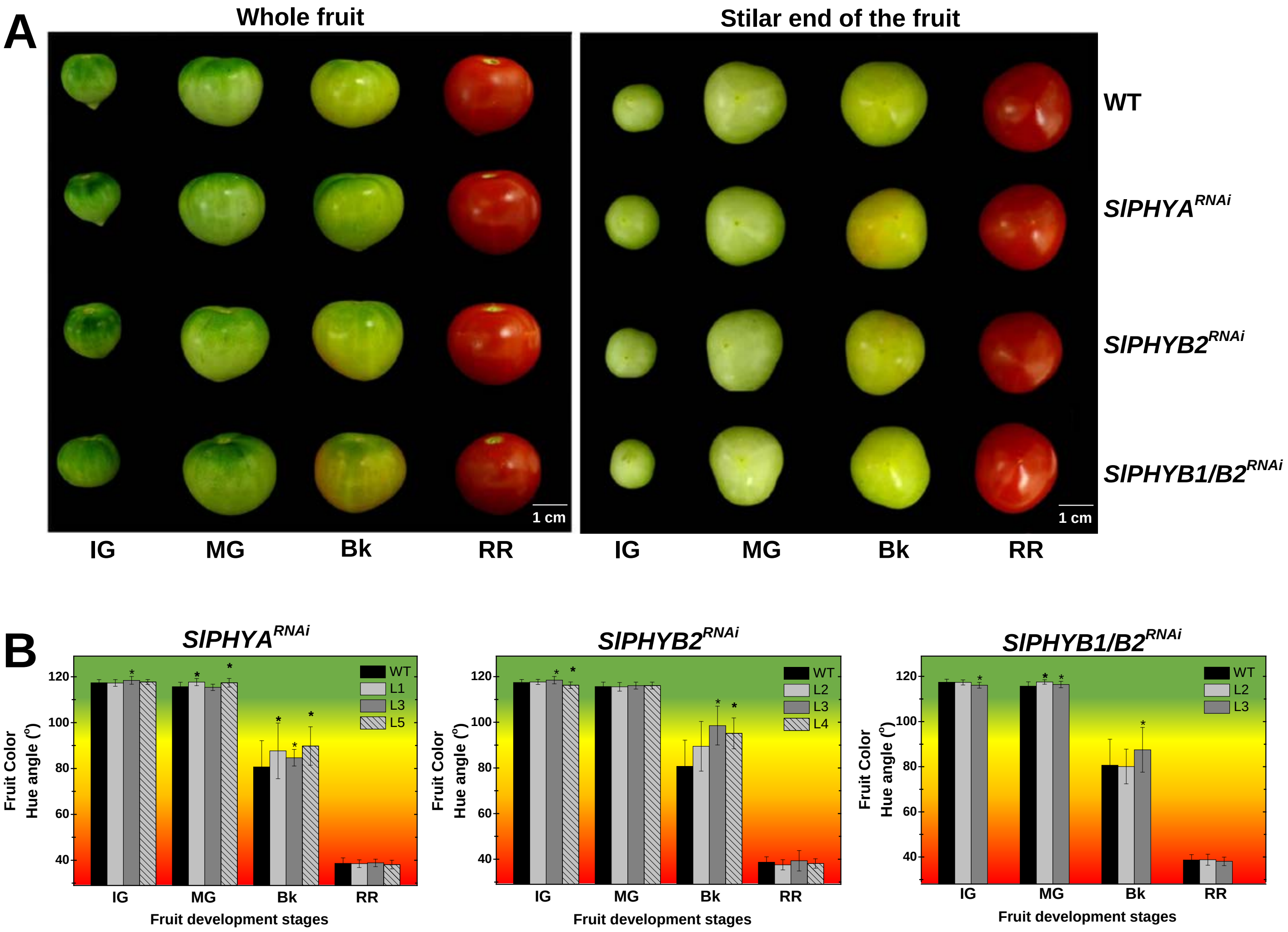

Figure S3. Visual phenotypes and color change of *PHY*-silenced fruits. (A) Representative fruits at each sampling stage. **(B)** Fruit color estimated by Hue angle. Data shown are mean ± SE of at least 16 biological replicates. Asterisks indicate statistically significant differences (Student's t-test, P<0.05) compared with the wildtype (WT) sample at each fruit development stage. The abbreviations indicate the following: IG, immature green; MG, mature green; Bk, breaker; RR, red ripe.

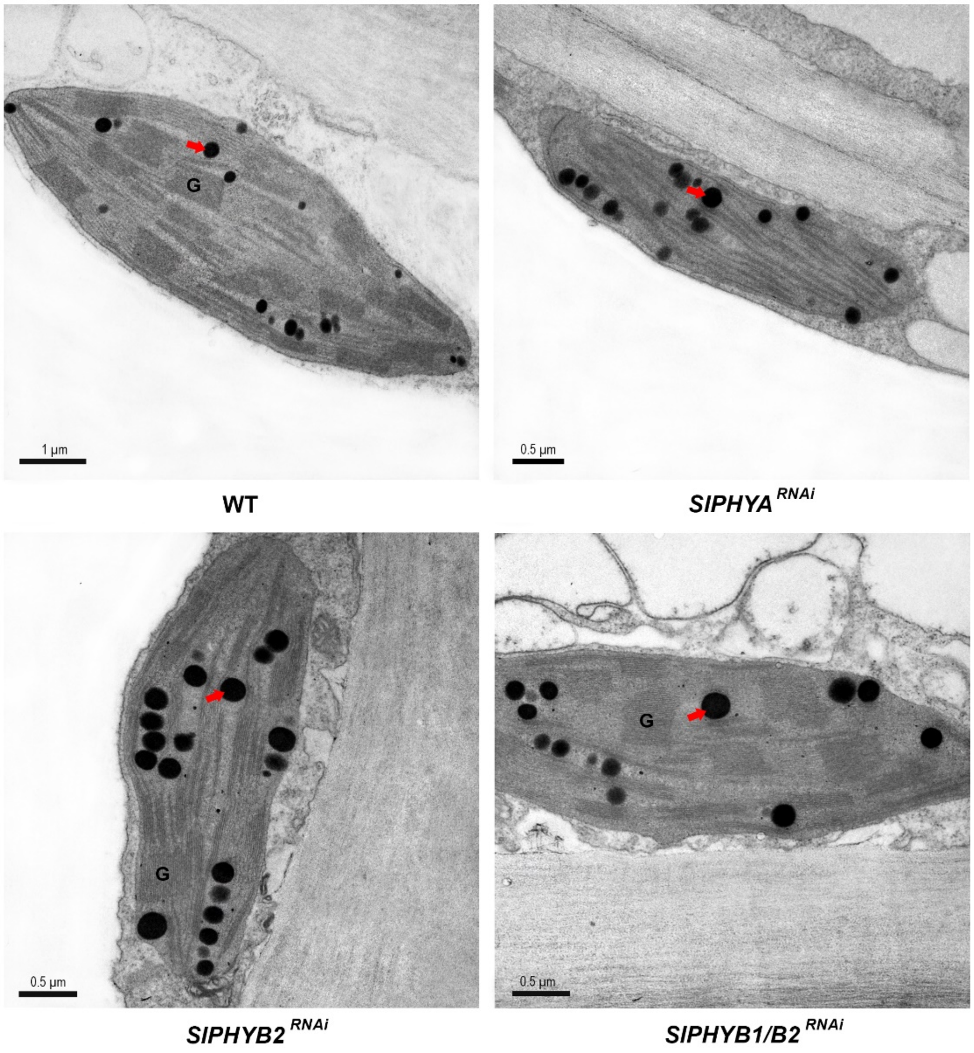

Figure S4. Plastid structure in *PHY*-silenced fruits. Representative transmission electron microscope images of plastids in immature fruits. Three fruits of each genotype were randomly picked for the ultrastructural analysis and two technical replicates taken at the pedicel region of each fruit. Arrows indicate plastoglobuli. G, granal thylakoid.

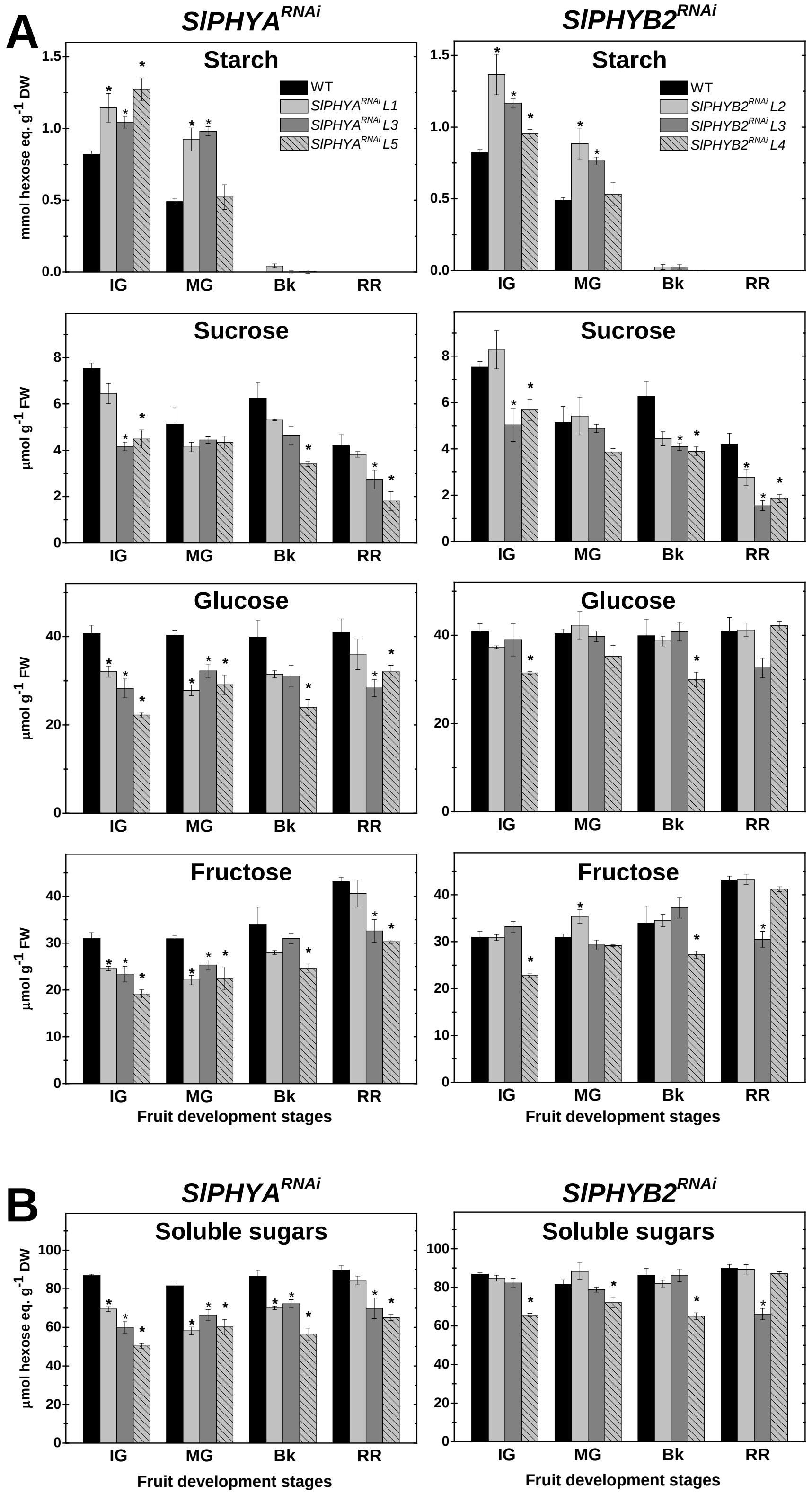

Figure S5. Carbohydrate profile in *PHY*-silenced fruits. (A) Starch, sucrose, glucose, and fructose content. **(B)** Summed values of the three soluble sugars analyzed (*i.e.* sucrose + glucose + fructose). Data shown are mean ± SE of at least three biological replicates. Asterisks indicate statistically significant differences (Student's t-test, P<0.05) compared with the wildtype (WT) sample at each fruit development stage. The abbreviations indicate the following: IG, immature green; MG, mature green; Bk, breaker; RR, red ripe.

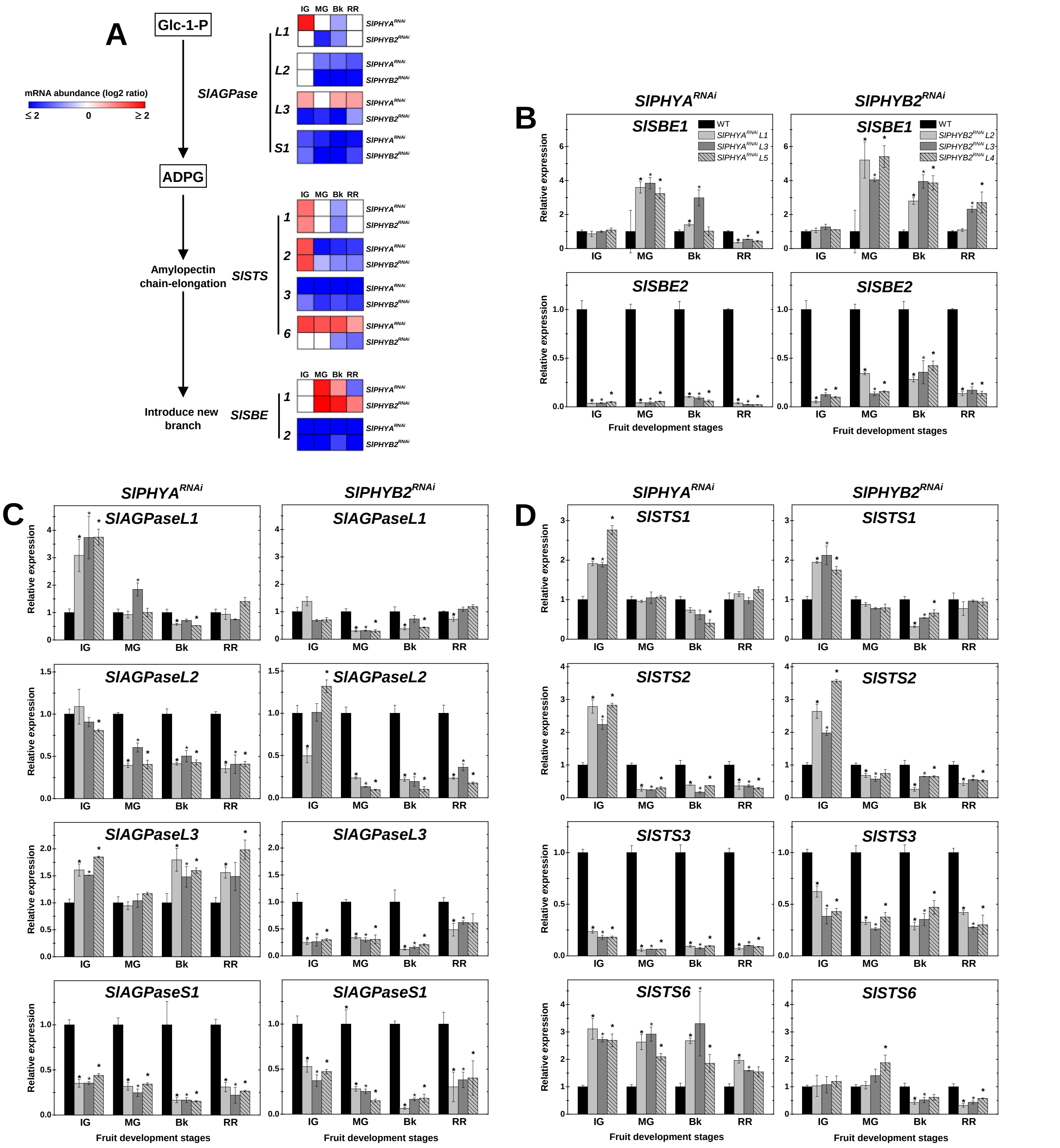

Figure S6. Transcript abundance of starch biosynthetic genes in *PHY*-silenced fruits. (A) Schematic representation of the major steps of starch biosynthesis and heatmap plot depicting the significant differences in mRNA levels of starch biosynthesis genes in *SlPHYA*^{RNAi} and *SlPHYB2*^{RNAi} lines compared with the wildtype (WT) sample (Student's t-test, P<0.05). For simplicity, the mean of the three transgenic line values is represented when at least two lines exhibited mean values statistically different from those detected in the WT. **(B)** Relative mRNA levels of tomato starch

branching enzyme (SlSBE)-encoding genes. **(C)** Relative mRNA levels of tomato genes encoding large (L1, L2 and L3) and small (S1) subunits of ADP-glucose pyrophosphorylase (SlAGPase). **(D)** Relative mRNA levels of tomato genes encoding starch synthase (SlSTS). Data shown are mean ± SE of at least three biological replicates. Transcript abundance was normalized against WT sample at each fruit developmental stage. Asterisks indicate statistically significant differences (Student's t-test, $P<0.05$) compared with the WT at each fruit development stage. The abbreviations indicate the following: IG, immature green; MG, mature green; Bk, breaker; RR, red ripe.

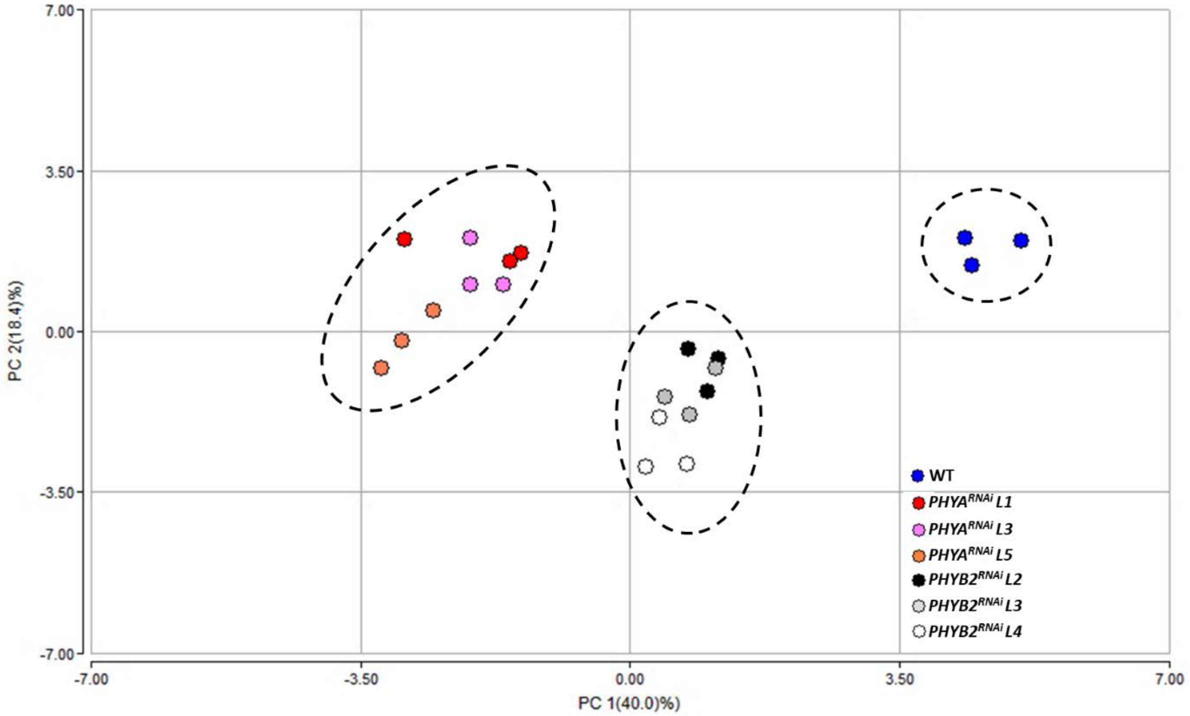

Figure S7. PCA of the expression profile of sink-related and starch biosynthesis-related genes. The principal component analysis (PCA) plot was generated with the transcript abundance of tomato genes encoding ADP-glucose pyrophosphorylase (SlAGPase), Starch synthase (SlSTS), Starch branching enzyme (SlSBE) and Invertase (SlLIN) in immature fruits of in the wildtype (WT) and in the *SlPHYA*^{RNAi} and *SlPHYB2*^{RNAi} transgenic lines.

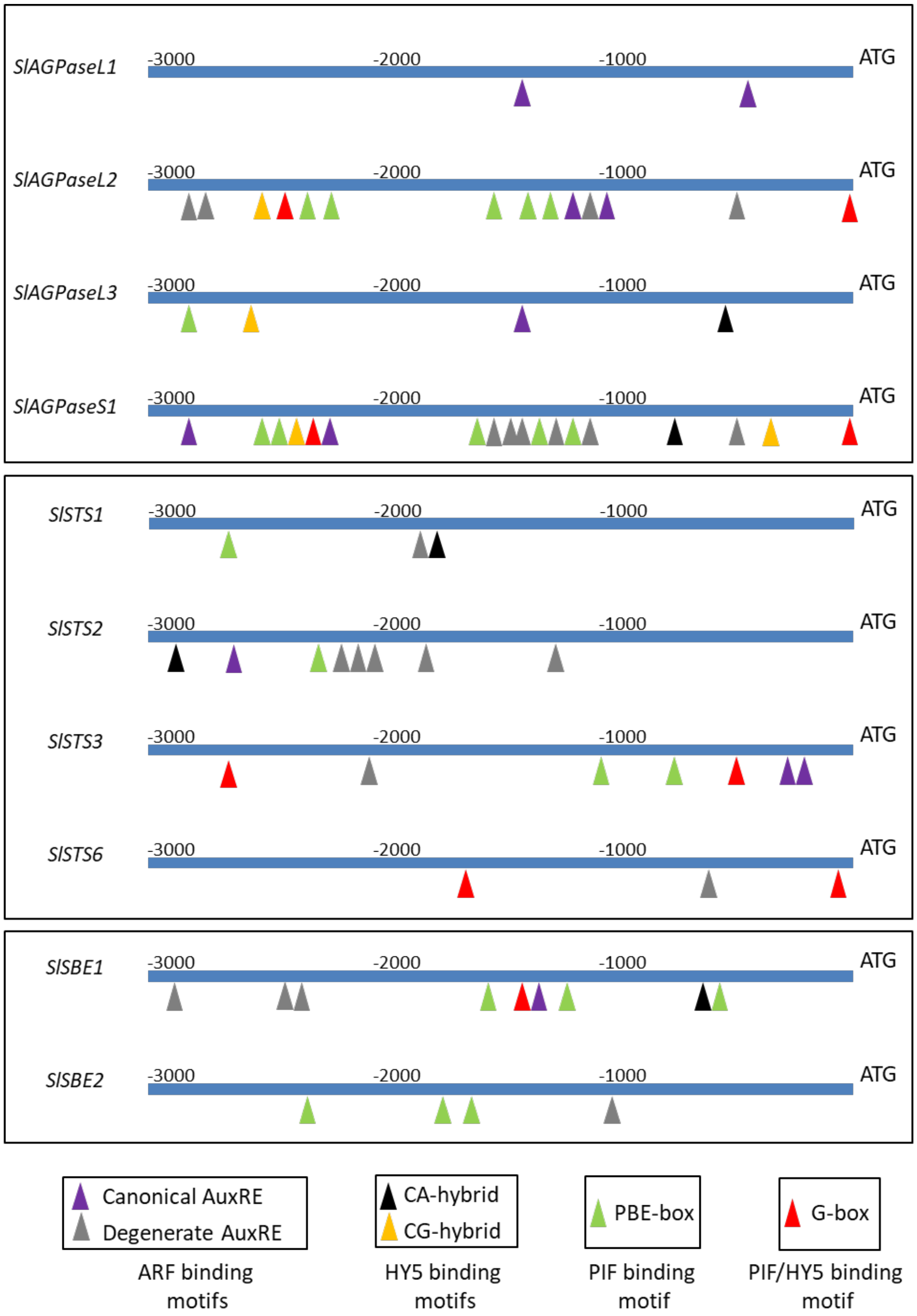

Figure S8. HY5-, PIF- and ARF-binding motifs identified in the promoter region of starch biosynthesis-related tomato genes. Fragments of 3 kb upstream the ATG initiation site of the tomato genes encoding ADP-glucose pyrophosphorylase small and large subunits (*SlAGPase*), Starch synthase (*SlSTS*) and Starch branching enzyme (*SlSBE*) are represented by a blue line. Motif positions are indicated by triangles: PBE-box (green), recognized by PHYTOCHROME-INTERACTING FACTORs (PIFs). CA-hybrid (black) and CG-hybrid (yellow), recognized by LONG HYPOCOTYL 5 (HY5). G-box (red), recognized by both PIFs and HY5. Canonical AuxRE (purple) and degenerated AuxRE (gray), recognized by AUXIN RESPONSE FACTORs (ARFs).

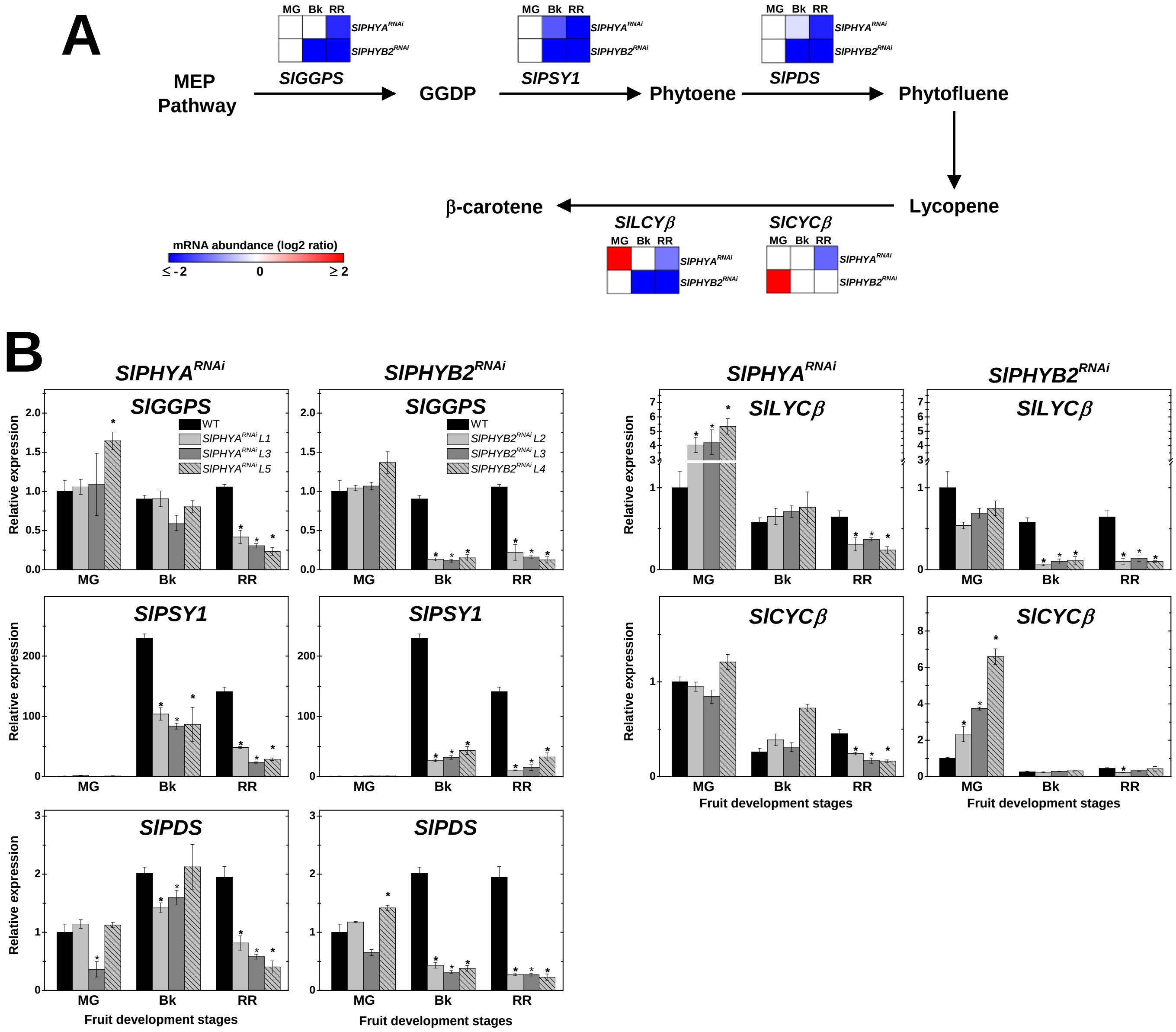

Figure S9. Carotenoid metabolism during ripening in *PHY*-silenced fruits. (A) Schematic representation of carotenoid biosynthetic pathway in tomato. Intermediate reactions were omitted. Heatmap represents statistically significant differences in mRNA levels of carotenoid biosynthesis genes in *SlPHYA*^{RNAi} and *SlPHYB2*^{RNAi} lines compared to the wildtype (WT) sample (Student's t-test, $P<0.05$). For simplicity, the mean of the three transgenic line values is represented when at least two lines exhibited mean values statistically different from those detected in the WT sample. **(B)** Relative mRNA levels of tomato genes encoding carotenoid biosynthetic enzymes. Data shown are mean ± SE of at least three biological replicates. Transcript abundance was normalized against the WT at each fruit developmental stage. Asterisks indicate statistically significant differences (Student's t-test, $P<0.05$) compared with the WT sample at each fruit development stage. The abbreviations indicate the following: MG, mature green; Bk, breaker; RR, red ripe, SlPSY, phytoene synthase; SlPDS, phytoene desaturase; SlLCYβ, chloroplast-specific β-lycopene cyclase; SlCYCβ, chromoplast-specific β-lycopene cyclase.

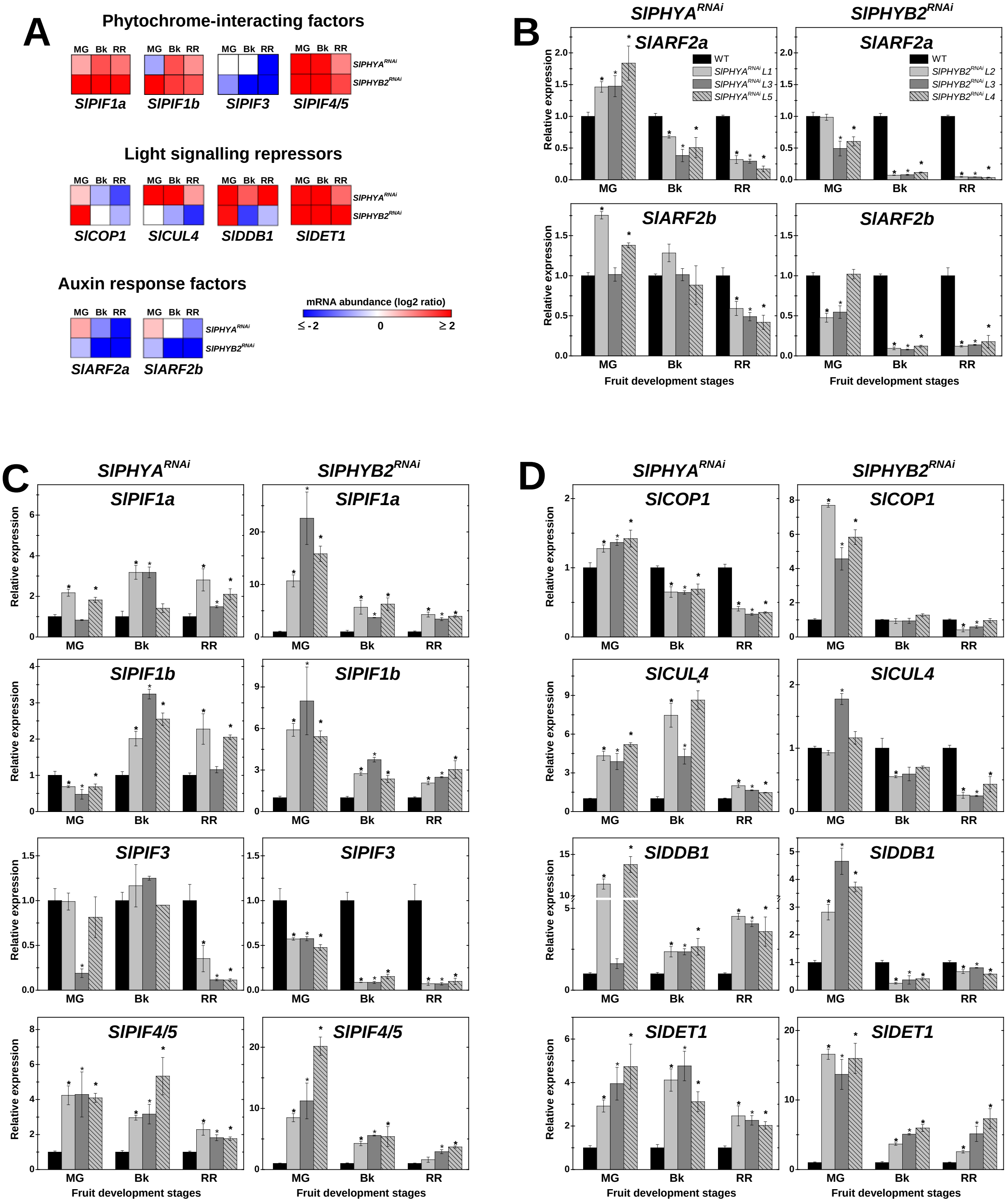

Figure S10. Transcript abundance of photomorphogenesis- and auxin-related genes in *PHY*-silenced fruits. **(A)** Heatmap represents statistically significant differences in mRNA levels of auxin and light signaling genes in *SlPHYA*^{RNAi} and *SlPHYB2*^{RNAi} lines compared to the wildtype (WT) (Student's t-test, P<0.05). For simplicity, the mean of the three transgenic line values is represented when at least two lines exhibited mean values statistically different from those detected in the WT sample. **(B)** Transcript abundance of the tomato *AUXIN RESPONSIVE FACTOR 2a* and *2b* (*SlARF2a* and *SlARF2b*) genes in PHY-silenced fruits. **(C)** Transcript abundance of tomato genes encoding

PHYTOCHROME INTERACTING FACTORs (SlPIFs). **(D)** Transcript abundance of tomato genes encoding the light signaling repressors CONSTITUTIVE PHOTOMORPHOGENIC 1 (SlCOP1), CULLIN 4 (SlCUL4), UV-DAMAGED DNA BINDING PROTEIN 1 (SlDDB1) and DE-ETIOLATED1 (SlDET1). Data shown are mean ± SE of at least three biological replicates. Transcript abundance was normalized against WT sample at each fruit developmental stage. Asterisks indicate statistically significant differences (Student's t-test, $P<0.05$) compared with the WT at each fruit development stage. The abbreviations indicate the following: MG, mature green; Bk, breaker; RR, red ripe; ARF, auxin response factor.

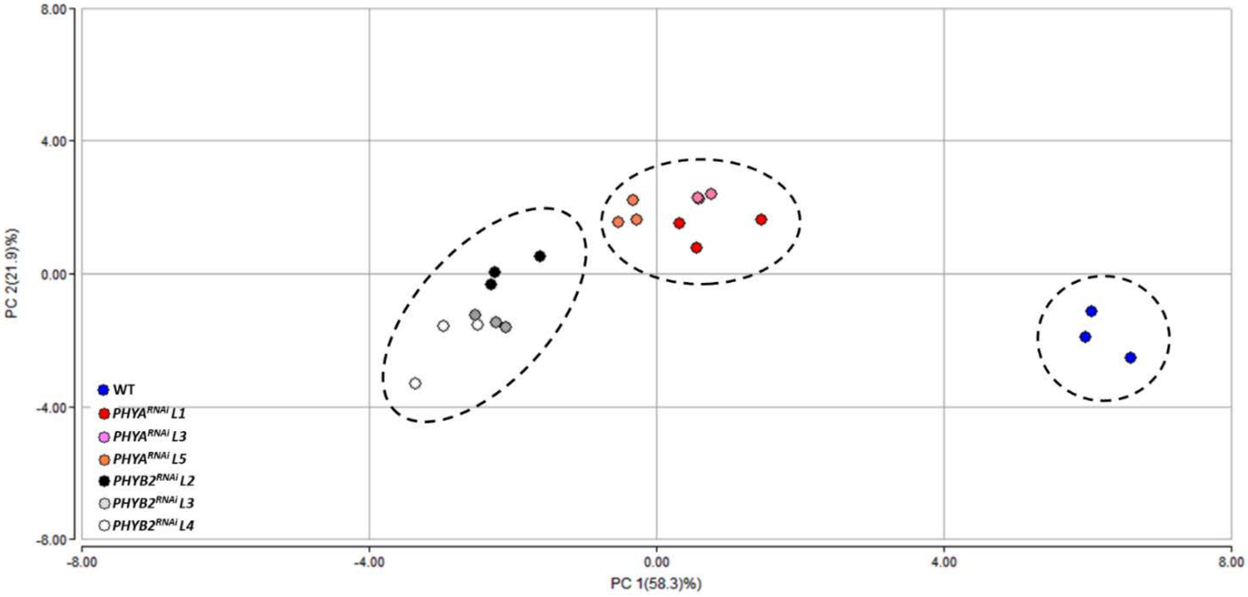

Figure S11. PCA of the expression profile of photomorphogenesis-related, auxin-related and carotenoid biosynthesis-related genes. The principal component analysis (PCA) plot was generated with the transcript abundance of tomato genes encoding key components of carotenoid biosynthesis pathway (SlGGPS, SlPSY1, SlPDS, SlLCYβ and SlCYCβ), positive regulators of carotenoids synthesis (SlARF2a and SlARF2b) and key components of light signaling transduction (SlPIF1a, SlPIF1b, SlPIF3, SlPIF4/5, SlCOP1, SlDDB1, SlDET1 and SlCUL4) in red ripe fruits of in the wildtype (WT), *SlPHYA*^{RNAi} and *SlPHYB2*^{RNAi} lines.

Table S1. Primer sequences.

Gene	Primer sequences[1]	Locus[2]
SlPHYA-RNAi	F: 5´- CACCATGTCGTCTTCAAGACCTAG - 3´/ R: 5´ - CGGTGGTAACTTTGTCTGACTTTGG -3´	Solyc10g044670
SlPHYB2-RNAi	F: 5´- CCACCCTTCAACTACCAATGCTGATTC - 3´/ R: 5´ - GTCCTGTTACAGTAGATGCTTC - 3´	Solyc05g053410
SlPHYB1/B2-RNAi	F: 5´- CACCATGGCTTCTGGAAGTAG - 3´/ R: 5´ - GTAGTTTTAACAGACTGTGAATAATC - 3´	Solyc01g059870
SlPHYA	F: 5´ - CACTCTCGTGGAGGATTCAT - 3´/ R: 5´ - GAGCCATAAAACACACACCC - 3´	Solyc10g044670
SlPHYB1	F: 5´ - ACTTCTGTTCGGTCCATTCC - 3´ / R: 5´ - TCTCAGACAACTGTGATGCC - 3´	Solyc01g059870
SlPHYB2	F: 5´ - GTGAGGGTTATTCAGGATGA - 3´ / R: 5´ - TGACCATATACTGAGGGTGAC - 3´	Solyc05g053410
SlPHYE	F: 5´ - CGCTATTGAGGAACCCACTT - 3´ / R: 5´ - GCATCAACACCAATCAGACC - 3´	Solyc02g071260
SlPHYF	F: 5´ - ACTAGCCAAGATCATTGACG - 3´/ R: 5´ - CTCCAAGATTGAACTCACAAG - 3´	Solyc07g04548
SlCOP1	F: 5´ -TTGCCCCTGTTGTTCTCA-3´ / R: 5´ - CCAATGAATGACGAAACTGTTC - 3´	Solyc12g005950
SlCUL4	F: 5´ - GGAGGAATTGGAGGGGACATTA -3´/ R: 5´- GAAATCATGGACTTTTCAGCATCA - 3´	Solyc02g021470
SlDDB1	F: 5´ - ATCTCGTGCCTGGACATAAA - 3´ / R: 5´ - CGAAGGAACACATCAGAACA - 3´	Solyc02g021650
SlDET1	F: 5´ - GCTATTGACCGCCATAGACA - 3´ / R: 5´ - TCTTAGTTCGCCCATCTGTG - 3´	Solyc01g056340
SlPIF1a	F: 5´- AACTTCTTGCTTTGCTCTCTG - 3´/R: 5´- GCTCCGCCCATAAATCA - 3´	Solyc09g063010
SlPIF1b	F: 5´-TAGTATGGCAAAATGGTGGAG - 3´ /R: 5´- CGGCGTCACAACTCGGTG - 3´	Solyc06g008030
SlPIF3	F: 5´- AAGGCTTCCCAATAATGC - 3´ /R: 5´- CCATCAGACCAAACTTCCC - 3´	Solyc01g102300
SlPIF4/5	F: 5´- GGCTTAGGTTCACATACAG - 3´ /R: 5´- TGATGGTGTCGTTGTCTC - 3´	Solyc07g043580
SlGLK2	F: 5´- ATGTTTGGGGGCATCCACATG -3´/R: 5´- GCAAATCAGAGGCAACTGTGTC - 3´	Solyc10g008160
SlARC3	F: 5´GGAAGTGATGATAGAGTCGT - 3´/ R: 5´- GCTGAATCTGGAATCTTGCTGTC - 3´	Solyc04g017620
SlARC6	F: 5´- GCTTACGGCAGTTCTGTG - 3´/R: 5´- CGTTGTCGTCCTTTGAGTG - 3´	Solyc04g081070
SlFTsZ1	F: 5´- AGCAGCCGAACAAGCAACTC - 3´/ R: 5´- CTGAAACGACTGGGTGAAAC - 3´	Solyc07g065050
SlFTsZ2	F: 5´- GAGCAGTTCAAGCCCAAGAAG - 3´/R: 5´ - CACGCACATCAGCAAAATCC - 3´	Solyc09g009430
SlPDV2	F: 5´ - TCTTGACTTTGGCTGGGTTC - 3´/R: 5´- TTCACGACGCACTGAGTTTC - 3´	Solyc03g007980
TRR 3/4	F: 5´ - CCAACATCACCAACAAGACG - 3´ / R: 5´ - ATGGGAGAGGAGGGCTTAAA - 3´	Solyc05g006420
TRR 7/15	F: 5´ ATGTTTGGAGGAAGGTGCTG - 3´ / R: 5´ - TGATTGTGTCGTTGGATCGT - 3´	Solyc03g113720
TRR 8/9a	F: 5´ - TGCTTAGAAGAAGGGGCAGA - 3´ / R: 5´ - GGGGGCTTTTACATTTGGTT - 3´	Solyc10g079600
TRR 8/9b	F: 5'- CAGCAGCAACAACAACAACAG - 3' / R: 5' - TCTGCCCAGCCCTTCTTAGATC -3'	Solyc02g071220
TRR 16/17	F: 5´ - GGTCTAAGGGCGTTGGAGTA - 3´ / R: 5´ - ATCTGAGCCCCTTCTTCCAT - 3´	Solyc06g048930
SlCRF1	F: 5´ - CTGACGCCGTTACCAATTTT - 3´ / R: 5´ - ACGTCGAAACCAAAAGCATC - 3´	Solyc06g082590
SlCRF2	F: 5´ - GAAGGGGAACAGGGGTTTTA - 3´ / R: 5´ - GCGGTCTTCTTCCTCCTCTT - 3´	Solyc08g081960
SlCRF3	F: 5´ - CGGACCATGACACAACAGAC- 3´ / R: 5´ - CTTACCCCATTTTCGTCGTC - 3´	Solyc01g008890
SlCRF5	F: 5´ - CCTCTGTTCTCCCACCTCA- 3´ / R: 5´ - AAGGGGATCGAAAATCGAAG - 3´	Solyc01g095500
SlCRF9	F: 5´ - AGGAAATGGGGGAAATATGC- 3´ / R: 5´ - AGGACCAGGGCTCAAATTCT - 3´	Solyc03g119580
SlAGPaseL1	F: 5´ - AGCAGACTACTACCAAACAG - 3´ / R: 5´ - ATTCCAATCGGTACTTTCC - 3´	Solyc01g109790
SlAGPaseL2	F: 5´ - CTGAAATTATCCCTTCTGCTG- 3´ / R: 5´ - ACTTCACTGTTCCAATATCCTC - 3´	Solyc07g019440
SlAGPaseL3	F: 5´ - CGCGCTACTTCGTAATAACC - 3´ / R: 5´ - CCATCAATTCTCCATTGCA - 3´	Solyc01g079790
SlAGPaseS1	F: 5´ - TGTAAGATTCACCATTCCGT - 3´ / R: 5´ - TCTTCTATAATTGCTCCCTCTG - 3´	Solyc07g056140
SlSTS1	F: 5´- GCTCAAATGTCCAAACTTCAC - 3´/ R: 5´- TATGGTTCCTGAGTCCTATCTG - 3´	Solyc08g083320
SlSTS2	F: 5´- CGCTGATTGGATTTATTGGA - 3´/F: 5´ GGATTATGTCAACACCTTTCTG - 3´	Solyc03g083090
SlSTS3	F: 5´- AGGATATTGCGAACCATTTC - 3´ / R: 5´- AGCCTCATCATACTTCAATAC - 3´	Solyc02g071040
SlSTS6	F: 5´- AGTTGGGAGGGGATTCAGAC - 3´/R: 5´- ACTTGTTGCATCCTCTCGTG - 3´	Solyc02g088000
SlSBE1	F: 5´-GTGATCGGAGGAATGCTAATGT -3´/R: 5´- GAAGGTCGGGATTCGGAATC - 3´	Solyc09g009190
SlSBE2	F: 5´- TCAACTTCCACCCAAATAACAC - 3´/ R: 5´- ACCAAATTCCCAAGCATCAC - 3´	Solyc04g082400
SlLIN5	F: 5´ - TTGGAAGGGATTGAGAATCG - 3´ / R: 5´ - AATTCCAGCCCATCCTTTCT - 3´	Solyc09g010080
SlLIN6	F: 5´ - AACCCGCTATCTACCCGTCT - 3´ / R: 5´ - GGGCTTGATCCACTTACGAA - 3´	Solyc10g083290
SlLIN7	F: 5´ - TCTTGACTTTGGCTGGGTTC - 3´ R: 5´ - TTCACGACGCACTGAGTTTC - 3´	Solyc09g010090
SlGGPS	F: 5´- GCTGTTGGTGTCTTATATCGTG- 3´/ R: 5´- CTTCTCAATGCCATAAACGCTG - 3´	Solyc09g008920
SlPSY1	F: 5´- CGATGGTGCTTTGTCCGATAC- 3´/ R: 5´- CTCATCAACCCAACCGTACC - 3´	Solyc03g031860
SlPDS	F: 5´- CGTTCCGTGCTTCTCCGC- 3´/ R: 5´- CTAGAACATCCCTTGCCTCCAG - 3´	Solyc03g123760
SlLYCβ	F: 5´- TTGACTTAGAACCTCGTTATTGG - 3´/ R: 5´- AACAGTTCCCTTTGTCATTATCT - 3´	Solyc04g040190
SlCYCβ	F: 5´- GCACCCACATCAAAGCCAGAG - 3´/ R: 5´- GCCACATGGAGAGTGGTGAAG - 3´	Solyc06g074240
SlARF2a	F: 5´ - GCAAGGTCAAGAGTTATCGA - 3´ / R: 5´ - CATTGGTTTCTCAGACAAGTC - 3´	Solyc03g118290
SlARF2b	F: 5´ - CTGGGTTAAGCGACAAGCTC - 3´ / R: 5´ - CCCCGCATTTGATACAGAGT - 3´	Solyc12g042070
SlARF4	F: 5´ - TGAAAGCCATCAACTCTCGG - 3´ / R: 5´ -ATCCCATCTGACCATCAAGCATC - 3´	Solyc11g069190
CAC (constitutive)	F: 5´ - CCTCCGTTGTGATGTAACTGG - 3´ / R: 5 -ATTGGTGGAAAGTAACATCATCG - 3´	Solyc08g006960
Expressed (constitutive)	F: 5´ - GCTAAGAACGCTGGACCTAATG - 3´ / R: 5´ - TGGGTGTGCCTTTCTGAATG - 3´	Solyc07g025390

[1]F: forward, R: reverse, [2]Locus according to the Sol Genomics Network database (http://solgenomics.net/).

Table S2. Homology of the RNAi fragments. (A) Percentage of sequence identity with *SlPHY* coding sequences. **(B)** Maximum length (nt) of perfect off-target matches (bold terms indicate the target genes for each RNAi fragment).

(A) Identity (%)

Silencing fragment	*SlPHYA*	*SlPHYB1*	*SlPHYB2*	*SlPHYE*	*SlPHYF*
SlPHYA^{RNAi}	100	45	45	42	53
SlPHYB2^{RNAi}	36	28	100	23	31
SlPHYB1/B2^{RNAi}	45	100	72	57	43

(B) Off-target match length (nt)

Silencing fragment	*SlPHYA*	*SlPHYB1*	*SlPHYB2*	*SlPHYE*	*SlPHYF*
SlPHYA^{RNAi}	**206**	5	5	9	12
SlPHYB2^{RNAi}	5	3	**160**	3	6
SlPHYB1/B2^{RNAi}	6	**205**	**22**	15	7

Table S3. Relative transcript ratios of tomato *AGPase* genes in immature fruits.

		SlAGPaseS1	*SlAGPaseL1*	*SlAGpaseL2*	*SlAGPaseL3*
**WT**		1.00 ± 0.15	**0.33 ± 0.08**	**0.09 ± 0.01**	**0.06 ± 0.01**
SlPHYA^{RNAi}	L1	1.00 ± 0.07	**0.65 ± 0.05**	**0.21 ± 0.02**	**0.15 ± 0.01**
	L3	1.00 ± 0.21	1.01 ± 0.21	**0.24 ± 0.05**	**0.20 ± 0.04**
	L5	1.00 ± 0.08	**0.64 ± 0.06**	**0.13 ± 0.01**	**0.17 ± 0.01**
SlPHYB2^{RNAi}	L2	1.00 ± 0.36	1.40 ± 0.35	**0.02 ± 0.00**	**0.18 ± 0.04**
	L3	1.00 ± 0.21	1.53 ± 0.33	**0.11 ± 0.02**	**0.29 ± 0.06**
	L4	1.00 ± 0.09	**1.48 ± 0.13**	**0.07 ± 0.00**	**0.22 ± 0.02**

Values represent means ± SD from at least three biological replicates and are expressed as relative transcript amount compared to *SlAGPaseS1* for each genotype. Statistically significant differences in the transcript ratio between each *SlAGPase* and *SlAGPase1* of each genotype are indicated in bold terms (Student's t-test, P<0.05).

Table S4. Carotenoid profile in red ripe fruits.

		Lycopene	Phytoene	Phytofluene	β-carotene	Lutein	Total carotenoids
**WT**		200.0 ± 7.1	6.88 ± 0.08	2.70 ± 0.04	1.09 ± 0.05	1.85 ± 0.06	212.4 ± 7.1
SlPHYA^{RNAi}	L1	**151.9 ± 11.9**	**9.26 ± 0.23**	**3.55 ± 0.14**	**0.81 ± 0.07**	**1.63 ± 0.02**	**167.1 ± 12.0**
	L3	**145.3 ± 11.6**	8.29 ± 1.26	3.10 ± 0.21	**0.53 ± 0.06**	**1.23 ± 0.17**	**157.3 ± 11.5**
	L5	**140.1 ± 17.0**	6.71 ± 0.50	3.22 ± 0.15	0.75 ± 0.18	**1.55 ± 0.07**	**152.5 ± 17.4**
SlPHYB2^{RNAi}	L2	**159.1 ± 11.9**	**15.28 ± 1.10**	**5.19 ± 0.40**	**0.06 ± 0.11**	**1.32 ± 0.21**	**181.5 ± 11.3**
	L3	**158.0 ± 14.0**	8.14 ± 0.78	2.90 ± 0.08	1.01 ± 0.01	1.76 ± 0.13	**171.8 ± 4.6**
	L4	**166.8 ± 6.4**	**8.60 ± 0.28**	3.55 ± 0.32	1.13 ± 0.07	1.71 ± 0.06	**175.9 ± 2.5**

Statistically significant differences in carotenoid content compared with the wildtype (WT) sample are indicated in bold terms (Student's t-test, $P<0.05$). Data shown are mean ± SE of at least three biological replicates.

Table S5. Antioxidant activity and total phenolics in red ripe fruits.

		Lipophilic antioxidant activity (µmol Trolox g^{-1} DW)	Hydrophilic antioxidant activity (µmol Trolox g^{-1} DW)	Total Phenolics (µmol gallic acid eq. g^{-1} DW)
**WT**		31.25 ± 0.81	250.72 ± 19.73	0.36 ± 0.01
SlPHYA^{RNAi}	L1	33.44 ± 3.02	243.28 ± 11.37	0.36 ± 0.04
	L3	31.96 ± 0.97	275.24 ± 0.31	**0.41 ± 0.01**
	L5	38.22 ± 4.97	268.52 ± 19.54	0.37 ± 0.01
SlPHYB2^{RNAi}	L2	30.89 ± 2.29	**301.82 ± 35.08**	**0.52 ± 0.07**
	L3	26.79 ± 4.08	**347.23 ± 44.39**	**0.48 ± 0.10**
	L4	**36.43 ± 3.28**	**316.47 ± 8.80**	**0.52 ± 0.05**

Statistically significant differences compared with the wildtype (WT) sample are indicated in bold terms (Student's t-test, *P*<0.05). Data shown are mean ± SE of at least three biological replicates.
